# Distribution and population genetic variation of cryptic species of the Alpine mayfly *Baetis alpinus* (Ephemeroptera: Baetidae) in the Central Alps

**DOI:** 10.1186/s12862-016-0643-y

**Published:** 2016-04-12

**Authors:** Marie Leys, Irene Keller, Katja Räsänen, Jean-Luc Gattolliat, Christopher T. Robinson

**Affiliations:** Department of Aquatic Ecology, EAWAG, Swiss Federal Institute of Aquatic Science and Technology, Ueberlandstrasse 133, CH-8600 Dübendorf, Switzerland; Institute of Integrative Biology, ETH-Zürich, CH-8092 Zürich, Switzerland; Department of Clinical Research, University of Bern and Swiss Institute of Bioinformatics, CH-3010 Bern, Switzerland; Musée cantonal de Zoologie, Palais de Rumine, Place de la Riponne 6, CH-1014 Lausanne, Switzerland; Department of Ecology and Evolution, University of Lausanne, Biophore, CH -1015 Lausanne, Switzerland

**Keywords:** Alpine biodiversity, Cryptic species, Nuclear microsatellites, Mitochondrial DNA, Sympatry

## Abstract

**Background:**

Many species contain evolutionarily distinct groups that are genetically highly differentiated but morphologically difficult to distinguish (i.e., cryptic species). The presence of cryptic species poses significant challenges for the accurate assessment of biodiversity and, if unrecognized, may lead to erroneous inferences in many fields of biological research and conservation.

**Results:**

We tested for cryptic genetic variation within the broadly distributed alpine mayfly *Baetis alpinus* across several major European drainages in the central Alps. Bayesian clustering and multivariate analyses of nuclear microsatellite loci, combined with phylogenetic analyses of mitochondrial DNA, were used to assess population genetic structure and diversity. We identified two genetically highly differentiated lineages (*A* and *B*) that had no obvious differences in regional distribution patterns, and occurred in local sympatry. Furthermore, the two lineages differed in relative abundance, overall levels of genetic diversity as well as patterns of population structure: lineage *A* was abundant, widely distributed and had a higher level of genetic variation, whereas lineage *B* was less abundant, more prevalent in spring-fed tributaries than glacier-fed streams and restricted to high elevations. Subsequent morphological analyses revealed that traits previously acknowledged as intraspecific variation of *B. alpinus* in fact segregated these two lineages.

**Conclusions:**

Taken together, our findings indicate that even common and apparently ecologically well-studied species may consist of reproductively isolated units, with distinct evolutionary histories and likely different ecology and evolutionary potential. These findings emphasize the need to investigate hidden diversity even in well-known species to allow for appropriate assessment of biological diversity and conservation measures.

**Electronic supplementary material:**

The online version of this article (doi:10.1186/s12862-016-0643-y) contains supplementary material, which is available to authorized users.

## Background

Historically, fixed morphological differences have been used to categorize the diversity of life and morphological traits remain important in taxonomy. However, phenotypic differentiation does not always correlate with genetic diversification. On the one hand, phenotypic plasticity can lead to pronounced phenotypic differentiation despite genetic similarity [1]. On the other hand, speciation without morphological divergence may occur, for instance, when mate choice is based on non-visual (e.g., chemosensory, acoustic) cues or when selection promotes morphological stasis [2].

Following the introduction of molecular techniques, it has become clear that a substantial proportion of biological diversity is morphologically hidden: many morphologically delimited species (so called morphospecies) consist of distinct evolutionary lineages that show varying levels of adaptive divergence and reproductive isolation [2]. In the most extreme case, multiple cryptic species may coexist in sympatry without interbreeding. Such cryptic species complexes are more widely distributed than previously thought, being reported for diverse taxonomic groups and across various ecosystems [3, 4].

Identifying and characterizing cryptic species, and the failure to do so, have far-reaching implications for basic and applied research. Critically, due to their distinct evolutionary histories, cryptic species may possess unique adaptations and evolutionary potential, and hence must be considered as separate evolutionarily significant units [5]. Ignoring the potential biological differences between cryptic species may render many biomonitoring, ecological risk assessment and conservation measures inappropriate [6–9].

The recognition of cryptic species, as well as their evolutionary histories and ecological requirements, is also crucial for predicting how climate change affects biodiversity [7, 10]. Climate change impacts are particularly alarming in alpine landscapes due to drastic environmental changes following increased temperatures and glacial retreat [11]. In glacial headwaters, severe alterations in hydrology and temperature regimes are threatening ecologically specialized and often endemic freshwater organisms [12, 13] - even without acknowledging cryptic diversity [10].

Importantly in this context, cryptic species are commonly reported in freshwater invertebrates [9, 14–17], and the mayfly genus *Baetis* (Ephemeroptera) comprises particularly remarkable examples of hidden diversity. Thus far, population genetic and phylogenetic studies have reported cryptic species complexes within *B. bicaudatus* [18], *B. rhodani* [19], *B. vernus* [20] and *B. harrisoni* [21]. The presence of multiple cryptic lineages has also been suggested within *B. alpinus* [22–25], a widespread, abundant and eurythermal alpine mayfly. The importance of understanding mayfly diversity is further emphasized by their wide use as biological indicators in ecotoxicological studies, and assessments of habitat quality and restoration success (e.g., [26, 27]).

In this study, we investigated patterns of genetic diversity in *B. alpinus* across the Swiss Alps using both nuclear microsatellite and mitochondrial DNA (mtDNA) markers. First, we used the combination of these two types of markers to reliably identify putative cryptic lineages testing the hypothesis of lineage separation. We followed the unified species concept [28], considering as separate species groups that are clearly distinguishable based on their multi-locus genotypes, with no or few intermediates when they occur in sympatry [29]. DNA barcoding, which relies on a single marker (typically the mitochondrial Cytochrome Oxidase I), has been extensively used for species delineations in animals [30], including mayflies [31]. However, shortcomings of single-gene approaches, such as the presence of pseudogenes [32], introgression [33] or incomplete lineage sorting [34] stress the need of a broader strategy. In addition, many studies report a lack of congruence of evolutionary histories inferred from mtDNA and nuclear genomes (i.e., mito-nuclear discordance [35]), emphasizing the importance of multi-locus approaches for a reliable assessment of genetic relationships among populations and species. Secondly, we used the nuclear microsatellite markers for a more detailed assessment of the extent of reproductive isolation among the putative cryptic lineages (e.g., [36–39]) and the population genetic structure within lineages. The latter is of interest as it reflects behavioral, historical and demographic differences between cryptic lineages as well as patterns of gene flow within lineages [40, 41].

We sampled *B. alpinus* species group from multiple basins in the central Alps in Switzerland. We used both large scale (regional) and within basin (local) sampling, combined with population genetic analyses, to test the following predictions. First, we tested for the presence of cryptic species within *B. alpinus*. These would be evident as strongly distinct lineages in both mtDNA and nuclear microsatellite markers. Our molecular data confirm the presence of two genetically highly distinct groups within *B. alpinus* (henceforth called lineage *A* and *B*) as well as some individuals from the closely related and superficially morphologically similar *B. melanonyx*. To gain insight into putative differences among these *B. alpinus* lineages, we investigated their relative abundances, habitat affinities and differences in population genetic structure, and tentatively re-analyzed morphological variation of lineages *A* and *B*. In particular, we tested whether the different lineages i) are associated with different environments (here, glacier-fed *vs.* spring-fed streams) – indicating ecological differences arising from either differences in habitat preference or local adaptation, ii) differ in population genetic variation and population structure - reflecting potential differences in evolutionary history as well as population demography, and iii) exhibit morphological differences in traits used in taxonomic identification of species.

## Methods

### Study species

Mayflies (Ephemeroptera) have aquatic egg to larval stages, strictly associated to freshwater habitats, followed by two short-lived winged stages: the pre-reproductive subimago and the sexually mature imago. Mayflies spend the longest part of their life cycle as larvae, the development of which is strongly dependent on water temperature [42, 43]. The length of the (pre-)adult stages is often shorter than one day. In the imaginal stage, males form swarms in which females fly into to mate. Mayfly females die after egg laying and males soon after mating. Dispersal occurs during adult aerial flight along streams as well as passive (drift) dispersal of larvae [44]. Due to their particular life cycle and habitat requirements, mayflies possess a low dispersal potential [16].

The genus *Baetis* was divided into 11 species groups [45], with some of them recognized as subgenera or genera (e.g. [17]). Among these, *Baetis* group *alpinus* encompasses 11 species [46], all but *B. alpinus* s.s. and *B. melanonyx* having restricted spatial distributions or being considered as endemic (e.g. [47, 48]). In particular, three species of the *B. alpinus* group are reported from Switzerland, namely: *B. alpinus* (Pictet) s.s., *B. melanonyx* and *B. nubecularis. B. alpinus* s.s. is a widespread and abundant alpine mayfly of the Palearctic region, reported from the Iberian Peninsula to the Ukrainian Carpathians [43, 49]. *B. melanonyx* is also widely distributed in the West Palearctic from the Iberian Peninsula to Ukraine. *B. nubecularis* is restricted to a few localities in the Swiss and French Jura mountains [48].

*B. alpinus* larvae are eurythermal and found in swift-flowing stony streams between 200 and at least 2600 m above sea level (a.s.l.). After mating, similarly to other *Baetis* species, adult females usually fly upstream from the emergence site and oviposit underneath large, stable protruding rocks [50], where eggs develop for several weeks until they hatch [42]. The life cycle of *B. alpinus* is described as plastic with regard to emergence timing and voltinism (which can be tri-, bi-, uni- or semivoltine) [43, 51–54] and is thought to be environmentally influenced [55]. *B. melanonyx* occurs in rhithral streams mainly between 600 and 1400 m a.s.l. In syntopic populations of *B. alpinus*, larval abundance was observed to decrease with elevation [46, 56]. *B. melanonyx* is distinguished from *B. alpinus* by a set of morphological traits [57].

### Study area and sample collection

**Fig. 1:**
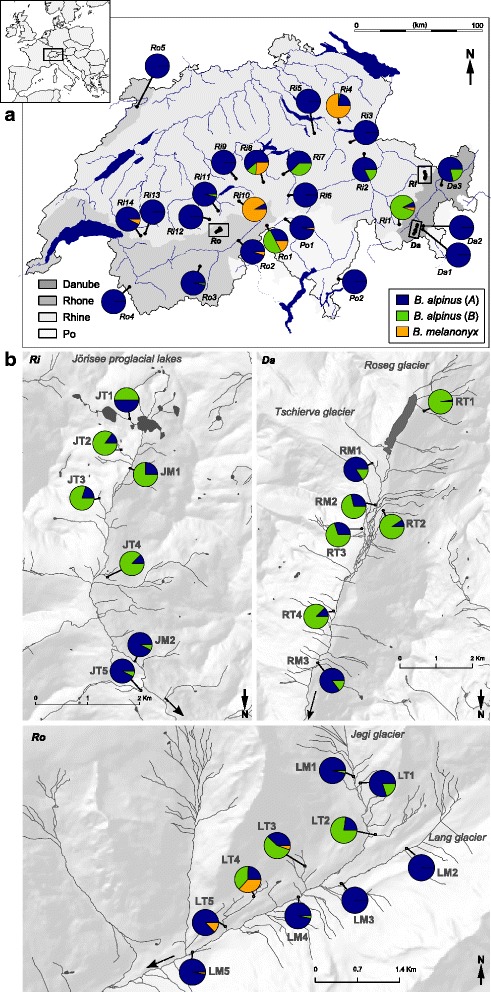
Sampling sites and the distribution of two *B. alpinus* cryptic lineages (*A* and *B*) and *B. melanonyx* within four major European drainages (Danube: *Da*, Rhone: *Ro*, Rhine: *Ri* and Po: *Po*) in the Swiss Alps. Sites are depicted at **a** the regional-scale and **b** within basin scale (labelled *Ri*, *Ro* and *Da* and by boxes in (**a**). In **b**, the first letter of the location name indicates the basin (J = Joerisee, R = Roseg, L = Loetschental) and the second letter the river type (M = main channel, T = tributary). Dotted arrows show river flow direction

This study investigated the genetic structure of *B. alpinus* at both regional and local scales. To examine regional patterns, 24 sites were sampled from multiple basins within each of four major European drainages in Switzerland (Rhine, Rhone, Danube and Po; Fig. 1a). The elevation of the sampling sites ranged from 476 to 2470 m a.s.l. To examine local-scale patterns, an additional 24 sites were sampled within three glacier-fed headwater basins in the Rhine (*Ri*), Rhone (*Ro*) and Danube (*Da*) drainages (Fig. 1b). In the local scale sampling, four or five spring-fed tributaries (T) and two to five glacial main-stream (M) sites were sampled along a longitudinal gradient downstream from the glacial snout (distance of sites from the glacier snout was min. 90 m and max. 6310 m, Fig. 1b). We chose this environmental contrast as *B. alpinus* is known to occur in both types of habitats and because main glacial streams and tributaries strongly differ in key ecological factors likely affecting performance of baetids. In particular, glacial streams exhibit colder but more fluctuating daily temperatures, higher stream-bed instability and more turbid waters relative to the more stable physico-chemical conditions of spring-fed streams [58, 59].

At each sampling site, mid to last-instar *Baetis* larvae were collected using a kick-net. Five kick-net samples were taken 3–5 m apart in an upstream direction with 40–50 larvae subsequently preserved in 96 % ethanol. For logistic reasons it was not feasible to taxonomically identify all individuals, but a few individuals per site were identified to the species level using taxonomic keys [57, 60]. Sampling was carried out from November 2011 to October 2012 and was completed the same day for all sites within a basin. Additional file 1: Table S1 provides additional information on the sampling locations.

### Molecular procedures

For genetic analyses, total genomic DNA was extracted from either complete larva or head capsules using a standard salting-out procedure [61].

#### Microsatellite genotyping

A total of 1591 individuals (average ± SD per site = 33.2 ± 9.0) were genotyped at 10 unlinked and polymorphic microsatellites, including both cDNA- (Ba_c1, Ba_c2, Ba_c3, Ba_c4) and genomic DNA-derived (gDNA, Ba_g1, Ba_g2, Ba_g3, Ba_g4, Ba_g5, Ba_g6) markers. The markers were developed by Ecogenics (Zürich, Switzerland) and are described in detail in the online supplement (Additional file 1: Table S2). cDNA-derived microsatellites were included because they tend to be more conserved, resulting in improved transferability across related species [62]. Interspecific transferability was considered important in this system where the presence of highly diverged lineages was expected based on previous work on *B. alpinus* and closely related taxa (e.g., [19, 23]). These markers also amplified DNA of *B. alpinu*s’ sister species, *B. melanonyx*.

Amplification by polymerase chain reaction (PCR) was performed in two separate multiplex reactions (Additional file 1: Table S2). PCR mixtures included 1 μL of DNA (at unknown concentration), 5 μL of QIAGEN Multiplex PCR Master Mix, and ddH_2_O to make up a total reaction volume of 10 μL. Primers were used at different concentrations to achieve similar signal intensities at the different loci (Additional file 1: Table S2). PCR cycling conditions were as follows: 15 min of denaturation at 95 °C, followed by 30 cycles at 94 °C (30 s), 56 °C (90 s), and 72 °C (90 s), followed by a final elongation step of 10 min at 72 °C. After dilution of the PCR products, fragments were separated using an ABI 3730xl DNA Analyzer (Applied Biosystems) and alleles were identified according to the PCR product size relative to a size standard (GeneScan™LIZ500; Applied Biosystems). The electropherograms were analyzed and manually edited using GENEMAPPER 3.7 software. To minimize the rate of genotyping errors, a second round of PCR and electrophoresis was performed for individuals with dubious multilocus genotypes (i.e., those with missing data or displaying rare alleles).

#### mtDNA COI sequencing

In a subset of 44 individuals, we sequenced a part of the Cytochrome Oxidase subunit I (COI) to determine the degree of mtDNA divergence among the cryptic species identified based on microsatellite markers (see Results), and to facilitate the comparison of our results with those of Finn et al. [22, 23]. The specimens were selected according to their geographic origin and cryptic species assignment (see Results). The primers LCO1490 and HCO2198 [63] were used to amplify a 658 bp fragment. Reactions contained 5 μl of DNA (unknown concentration), 12.5 μl KAPA2G HiFi HotStart Ready Mix (Kapa biosystems), 1.25 μl of each primer (10 μM) adjusted to a final volume of 25 μl with ddH_2_O. Initial denaturation (3 min at 95 °C) was followed by 40 cycles of 15 s at 94 °C, 15 s at 48 °C, 15 s at 72 °C, and a final extension of 72 °C for 1 min following Kapa standard instructions. PCR products were purified using the Wizard SV96 standard protocol (Promega) prior to sequencing (Microsynth, Balgach, Switzerland). When low concentration amplicons were obtained, the amplification was repeated twice, and both reactions were pooled in the purification step. Sequences were edited using CODONCODEALIGNER 5.1.5 (CodonCode Corporation, Centerville, MA, USA) and aligned using ClustalW in BIOEDIT 7.2.5 [64].

### Statistical analysis

#### Characterisation of microsatellite markers

All microsatellite loci were tested for variability and departures from Hardy-Weinberg equilibrium (HWE) within samples expected to be panmictic (i.e., from both the same location and genetic group defined here-after as "population"; see clustering analysis below). All HWE tests were performed with a minimum sample size of eight individuals using the randomisation procedure implemented in FSTAT 2.9.3.2 [65]. Linkage Disequilibrium (LD) between pairs of loci was assessed in GENEPOP 4.0.1.1 [66]. Using the same software, the frequency of null alleles was estimated according to Dempster et al. [67]. All loci were found to be highly polymorphic in all *B. alpinus* populations, and we found no consistent deviations from HWE or deviations from linkage equilibrium. In contrast, *B. melanonyx* showed high probabilities of null alleles for some site-locus combinations together with significant deviation from HW genotypic proportions. We therefore refrained from any further detailed population genetic analysis of this species. Detailed information on the number of alleles, observed and expected heterozygosities, and estimated null allele frequencies is provided in Additional file 1: Tables S4 and S5.

#### Cryptic lineage delimitation

For the microsatellite markers, cryptic species were identified in accordance with the ‘genotypic cluster’ definition, within the unified species concept framework [28]. Under this definition, distinct genotypic groups are considered as separate species when they show no or very limited interbreeding upon contact and, as a consequence, follow largely independent evolutionary trajectories [29].

The Bayesian clustering approach of STRUCTURE 2.3.4 [68] was used to identify genetic substructure within the full dataset and assign individual genotypes to specific genetic groups. We used an admixture model and correlated allele frequencies [69] without any prior information on the putative population affiliation of individuals and with 2x10^6^ Markov chain Monte Carlo (MCMC) iterations following a burn-in period of 1x10^5^ iterations. The likelihood of different numbers of genetic clusters (*K*) was assessed for *K* = 1–30 using 30 runs per *K*. The *ad hoc* statistic *ΔK* [70] was calculated and further visual exploration of the results was performed (see [71]) to determine the most likely value of *K*. Similarity coefficients between runs and the average matrices of individual membership proportions were estimated using CLUMPP 1.1.2 [72]. As multimodality was observed for all *K* (Additional file 2), the group of solutions most frequently observed among the 30 replicates was averaged and used for interpretation. Clusters were visualized using DISTRUCT 1.1 [73].

On the same data, we also performed a Discriminant Analysis of Principal Components (DAPC), a model-free multivariate method to identify genetic clusters, genetic clines and hierarchical structures [74]. The discriminant analysis was based on 60 principal components accounting for 87 % of the total genetic variation. The Bayesian Information Criterion (BIC) was used to determine the optimal number of clusters [74]. All analyses were conducted using *adegenet* for R.3.13 [75].

#### Congruence of mtDNA clades and nuclear genetic clusters

For the mtDNA markers, we created an alignment of the 44 COI sequences (including 41 *B. alpinus* and 3 *B. melanonyx*) produced in our study and 42 *B. alpinus* sequences from GenBank to gain insights into genetic variation across multiple mountain massifs within the range of *B. alpinus* (see Additional file 1: Table S3, [22, 23, 25]). We also included two *B. nubecularis* and one *B. melanonyx* unpublished sequences (provided by S. Rutschmann and M.T. Monaghan obtained as part of the FREDIE project) to gain insight into the extent of genetic divergence between *B. alpinus* cryptic lineages compared to taxonomically described species of the *B. alpinus* group. Using a fragment of 613 bp from all 89 sequences, 70 unique haplotypes were identified using the *haplotype* function in the *ape* package for R [76]. A maximum likelihood (ML) gene tree was inferred on all unique COI haplotypes using the software PhyML 3.01 [77] and the TN93 + G + I substitution model that best fit the data according to the Akaike information criterion (AIC). The evolutionary model selection was conducted using both the *phymltest* function of the R package *ape* and PhyML 3.01 software [76, 77]. Branch support was calculated with 500 bootstrap replicates. *Baetis rhodani* was used as an outgroup (published sequence, Additional file 1: Table S3). Pairwise distances assuming the Tamura-Nei nucleotide substitution model were computed using the *dist.dna* function of the *ape* package [76].

To derive mtDNA clades, we performed a Parsimony haplotype network analysis (Additional file 3). Next, a simple graph analysis using all unique COI haplotypes and the function *genegraph* implemented in the R package *adegenet* was completed [75].

#### Comparing the cryptic lineages in relative abundance and spatio-ecological attributes

We found two genetically strongly distinct *B. alpinus* lineages (henceforth called lineage *A* and *B*). A small subset of specimens (*N* = 112) identified as *B. melanonyx* were recovered as a monophyletic lineage. The relationship between the relative abundance of *B. alpinus* lineage *A* and *B* and spatio-ecological attributes (i.e., elevation, drainage and stream-type) was tested using generalized linear models with logit link and binomial error. These analyses were conducted separately at the regional and local scales. First, at the regional scale, we tested for the effect of elevation and drainage (fixed effects) on the relative abundance of the two lineages. To reduce bias due to differences in sampling design among basins that included regional *vs*. local scale data, one site was randomly selected within the *Ro*, *Ri* and *Da* basins for regional analyses (hence, data consisted of 27 sites). Secondly, at the local scale (i.e., 24 sites), we tested for the effects of stream-type (glacial main stream sites *vs*. spring-fed tributaries) and basin (*Ro*, *Ri* and *Da*) by including these as fixed factors in the model. As overdispersion was detected for both the regional and local models, we fit quasi-binomial models and used *F*-tests for model comparison [78]. Terms were dropped from the model in a stepwise manner if their removal did not lead to a significant increase in deviance. The generalized linear models were run using the library *stats* for R 3.1.3 [79].

#### *Population genetic structure within* B. alpinus *cryptic lineages*

To test for genetic substructure within lineage *A* and *B*, we used nuclear microsatellite markers and three complementary approaches: 1) Bayesian clustering (STRUCTURE), 2) multivariate ordination without considering the geographic origin of the samples (DAPC), and 3) multivariate ordination with spatial information (spatial Principal Component Analysis; sPCA [80]). Each individual was assigned to a cryptic lineage based on the maximum cluster membership probability estimated by DAPC as this method does not make any assumption with respect to mating system, population structure or allelic frequency models.

STRUCTURE and DAPC were run as described above, but we assessed only *K* = 1–10 in the STRUCTURE analysis. The sPCA complements the first two methods by explicitly identifying spatial patterns of genetic structuring (e.g., clines) across the landscape and accounting for spatial autocorrelation caused, for instance, by spatially limited mating or uneven distribution of sampling sites [81, 82]. We used the Gabriel graph to define which populations are neighbours in the sPCA algorithm. Following the methods in Jombart et al. [80], we tested for global (neighbouring populations are more similar than expected) and local (neighbouring populations are more dissimilar than expected) spatial structures using permutation tests with the number of axes retained based on the sPCA eigenvalue decomposition. If significant genetic structure was detected, we qualitatively identified spatial allele frequency patterns by visualizing the entity scores on a geographic map. The scores of the first two principal components were simultaneously represented using the RGB (red, green and blue) color model as in Menozzi et al. [83]. All analyses were performed using the *adegenet* package [80] for R 3.1.3 [79].

Population differentiation across all populations was assessed using classical *F*-statistics according to Weir and Cockerham [84]. A permutation test was used to determine whether *F*_ST_ values were significantly different from 0 for each locus and over all loci (10,000 randomisations of multilocus genotypes among populations). Further, we tested for an association between geographical and genetic distance between populations accounting for genetic substructure using a partial Mantel test based on three distance matrices: 1) Pairwise genetic distance (*F*_ST_) between populations of at least eight individuals, 2) pairwise Euclidean distance, and 3) a 0/1 matrix indicating if two populations belong to the same or different subgroups within cryptic lineages. To create this last matrix, we assigned the population as a whole to the subgroup where, averaged across all individuals, it had the highest membership probability. The significance of *r* values was tested with a partial Mantel test (10,000 permutations) performed in the package *ecodist* for R [79].

Genetic diversity within each *B. alpinus* lineage was examined based on all populations with at least eight individuals by calculating the total and mean number of alleles (*A*_*t*_ and *A*_*m*_, respectively), allelic richness (*A*_r_) and gene diversity (*H*_E_) in FSTAT 2.9.3.2 [65]. Allelic richness was estimated using the rarefaction approach proposed by El Mousadik and Petit [85] based on eight diploid individuals. Private allelic richness (*A*_r_*P*) was computed following the rarefaction procedure (*n* = 8) in the ADZE software [86]. Levels of genetic polymorphism (*A*_r_, *A*_r_*P*, and *H*_E_) were compared between *B. alpinus* lineages. Differences were tested using the Kruskal-Wallis Rank Sum test and the multiple comparison test implemented in the R package *pgirmess* [87].

### Morphological differences between cryptic lineages

The initial identification of the *B. alpinus* specimens was based on standard taxonomic criteria related to (in particular) mouth parts, legs and tergites of larvae [57]. Upon genetic identification of strongly distinct lineages, we selected a subset of samples for which genetic lineage was known for a tentative morphological screening to confirm whether the lineages were indeed morphologically indistinguishable. A detailed microscopic examination of morphological characters was conducted on a subset of 23 *B. alpinus* larvae (N_*A*_:12; N_*B*_:11). These specimens were selected according to their lineage and genetic subgroup identity, and collected from the same site (*LM3* in the *Ro* basin) when possible. As some genetic subgroups were only present either in eastern or western Switzerland, additional specimens belonging to genetic subgroups not present in *LM3* were selected in *Da.* Specimens, preserved in ethanol (96 %), were dissected using a stereo-microscope and mounted on slides in Canadian balsam after a short stay in Creosote solution. Morphological characters included: i) the number of spine-like setae at the apex of the maxillary palp, ii) the number of rows of long setae on the dorsal margin of the femora, iii) the presence and number of setae at the apex of the tarsus, iv) the triangular spines of the distal margins of tergum IV, v) the presence of micropores on tergum IV, vi) the presence and number of scales of tergum IV, and vii) the margins of the gills IV.

## Results

### Evidence for cryptic lineages

**Fig. 2:**
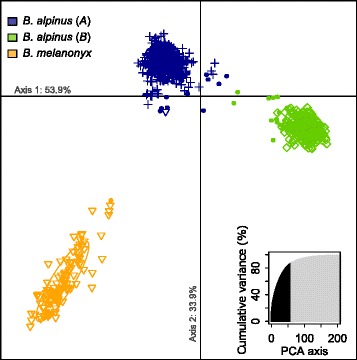
Clustering analysis of multilocus genotypes of 1591 individuals of *B. alpinus* species group based on 10 microsatellite markers. The DAPC scatterplot shows the first two principal components on the X and Y axes. Each colored dot represents an individual genotype assigned to one of the three clusters by DAPC (colors and ellipses), with symbols corresponding to each genetic group defined by Bayesian clustering analysis of STRUCTURE: *B. alpinus* lineage *A*, crosses; *B. alpinus* lineage *B*, diamonds; *B. melanonyx*, triangles. Filled dots indicate admixed individuals with a cluster membership coefficient <0.85 in the STRUCTURE analysis. The number of principal components retained and their cumulative variance explained are highlighted in black in the insert. The proportion of variance captured by the first and second DAPC axes is indicated on each axis

Bayesian clustering of the microsatellite data revealed three distinct genetic groups, of which two corresponded to *B. alpinus* and one to *B. melanonyx* (termed *A*, *B* and *B. melanonyx*; Fig. 2; Additional files 2 and 4). This grouping was strongly supported by concordant DAPC results, with nearly identical individual assignment (99.7 %) to the genetic groups identified by STRUCTURE (Fig. 2; Additional file 4). The magnitudes of differentiation, as indicated by between-lineage or species pairwise *F*_ST_s, were *F*_ST_ = 0.189 for *A vs B*, *F*_ST_ = 0.234 for *A vs B. melanonyx* and *F*_ST_ = 0.318 for *B vs B. melanonyx* (all *P* <0.001). The strong separation of the genetic clusters was due to both private alleles at some loci and allele frequency differences at all loci. A total of 1543 out of 1591 genotypes (97.0 %) could be clearly assigned to one of the three clusters (membership probability ≥0.85) and only 48 genotypes (3.0 %) showed admixed membership proportions.

**Fig. 3:**
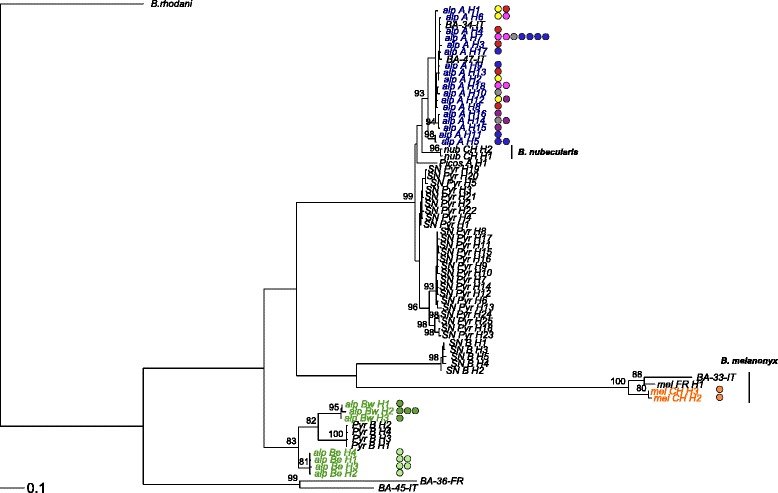
Maximum likelihood mtDNA gene tree of the *B. alpinus* species group COI unique haplotypes. The color of the sample label indicates the *B. alpinus* cryptic lineages and *B. melanonyx* as identified based on nuclear microsatellites (*A*: Blue; *B*: Green; *B. melanonyx*: Orange). *B. alpinus*, from Genbank and unpublished *B. melanonyx* and *B. nubecularis* sequences were included (in black). Additional information on the published or provided sequences, including the geographic origin of the samples, mountain massif and respective GenBank accession number, is provided in Additional file 1: Table S3. *Baetis rhodani* was used as an outgroup. Filled colored circles indicate nuclear subgroup membership for *B. alpinus* lineages *A* and *B* (see Fig. 6). Numbers at nodes represent branch support based on 500 bootstrap replicates, and are shown for support values >80 %. The scale bar indicates the estimated number of nucleotide substitutions per site

In total, 27 different haplotypes (25 *B. alpinus* and 2 *B. melanonyx*) were identified in our set of 44 mtDNA sequences. Our mtDNA sequences, together with those from GenBank, yielded a total of 70 haplotypes. In our data, the clustering observed based on mtDNA sequences matched that based on microsatellite genotypes (Fig. 3). Overall, haplotype Parsimony networks and genetic transitive clusters yielded similar results (Additional files 3 and 5). Mean sequence divergence, assuming the Tamura-Nei nucleotide substitution model, varied between 0.2–6.9 % within clades (1.2 and 4.5 % within lineages *A* and *B*, respectively) while genetic divergence among clades ranged from 4.9 to 27.1 % (18.9 % between *A* and *B* lineages; see Additional file 1: Table S4).

### Relative abundance and spatial distribution of the *B. alpinus* lineages and *B. melanonyx*

Both *B. alpinus* lineages occurred in several major European drainages (*B* was not found in the Po for which only few samples were available). However, the *B. alpinus* lineages showed pronounced differences in abundance: 1001 individuals were assigned to lineage *A* and 478 to *B,* indicating that *A* is much more abundant than lineage *B*. Some 112 individuals belonged to *B. melanonyx* which occurred only at eight sites. Mitochondrial DNA sequences revealed high genetic similarity of Swiss haplotypes of each lineage with those from other mountain ranges (central Apennines, Ligurian Apennines, Pyrenees and Sierra Nevada; see Fig. 3 and Additional file 1: Table S3), suggesting a wider European distribution of the lineages found in the Central Alps.

**Fig. 4:**
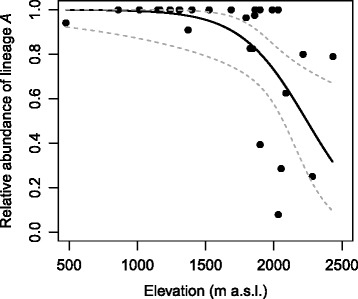
Relative abundance of *B. alpinus* lineage *A* versus lineage *B*. Fitted line of the optimal quasi-binomial model including elevation as the only explanatory variable. The grey dotted lines indicate 95 % confidence intervals

Spatial co-occurrence of the two *B. alpinus* cryptic lineages was common (Fig. 1), whereby both lineages were present in 29 of the 48 sites. At the regional scale, the relative abundance of lineages *A* and *B* showed a significant association with elevation, with lineage *B* being more abundant at higher elevations (Elevation: *F* = 12.87, *P* = 0.002, Drainage: *F* = 0.52, *P* = 0.673, see Additional file 1: Table S6; Figs. 1 and 4). At the local scale, lineages *A* and *B* differed in relative abundance in relation to stream-type, with lineage *B* being more abundant in groundwater-fed tributaries and *A* in glacier-fed main streams (Stream-type: *F* = 9.42, *P* = 0.006). There was also an association of lineages with particular drainages (Drainage: *F* = 4.66, *P* = 0.022, Additional file 1: Table S6; see Fig. 1b).

### Population genetic variation within the lineages

**Fig. 5:**
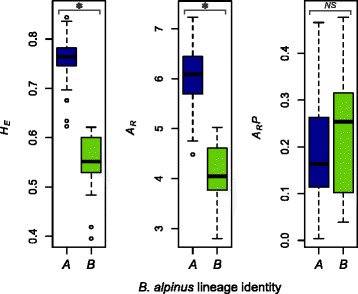
Genetic diversity estimates (expected heterozygosity *H*_E_, allelic richness *A*_r_ *,* and private allelic richness *A*_r_ *P*) for the two *B. alpinus* lineages *A* and *B*. Box-plots indicate the median (horizontal line), the 25^th ^and 75^th^ percentiles (bottom and top of each box), and the minimum/maximum values (vertical dashed lines). * = *P* < 0.01

In accordance with differences in relative abundance of the lineages, genetic diversity, as measured by *H*_*E*_ and *A*_*r*_, was higher in *B. alpinus* lineage *A* than in *B* (Fig. 5), while private allelic richness (*A*_*rP*_) did not differ significantly between the two lineages.

**Fig. 6:**
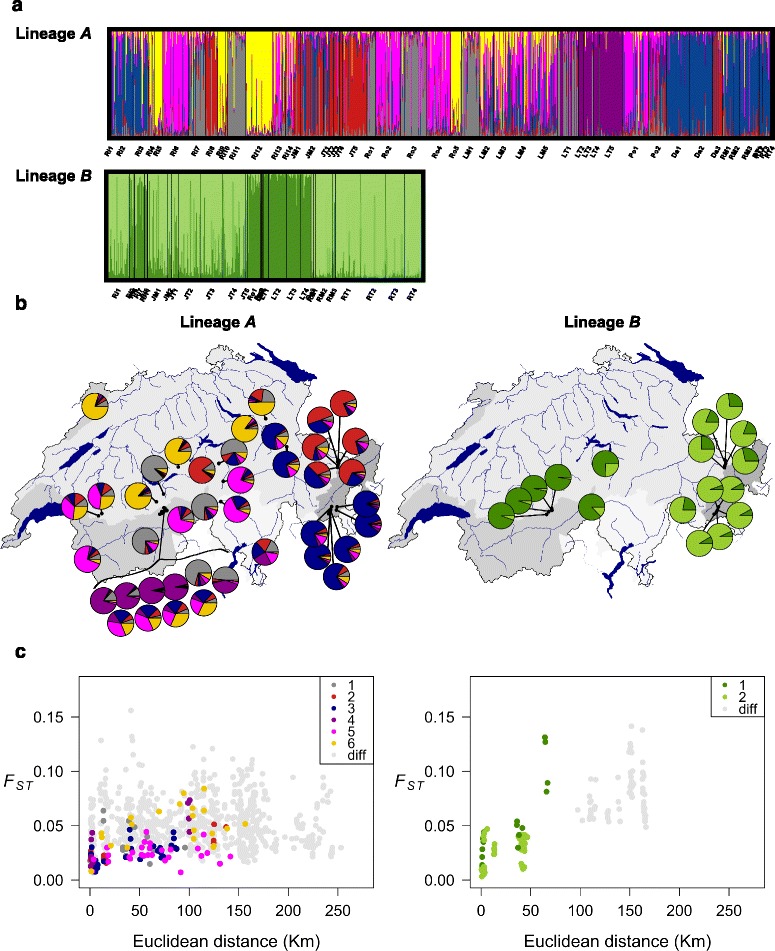
**a** Results from Bayesian clustering (STRUCTURE), **b** spatial distribution patterns of within lineage genetic groups and **c** relationship between genetic differentiation and geographical distance for the two *B. alpinus* lineages (*A* and *B*). **a** Bayesian assignment probabilities of *A* and *B* individuals into six and two inferred clusters, respectively. Each individual is represented by a vertical line (x axis) partitioned into *K* colored segments that represent the individual's estimated membership proportion in each of the *K* clusters (y axis). Sampling site labels (letters along the x axis) are identical to Fig. 1 (see also Additional file 1). **b** Membership probabilities averaged across all individuals per population for lineages *A* and *B*. The colors correspond to colors in panel (**a**). **c** Relationship between within-lineage pairwise population differentiation (*F*_ST_) and Euclidean distances. Pairwise population affiliation comprising identical or different sub-clusters is indicated with a number or "diff", respectively. Each population was assigned to the genetic cluster with the highest probability across individuals (e.g., the lineage *A* population in the top left corner of (**b**) would be assigned to yellow)

Further substructuring within the *B. alpinus* lineages was evident. For lineage *A*, Bayesian clustering indicated two peaks of *ΔK* at *K* = 3 and *K* = 6. Visual inspection of the STRUCTURE barplots indicated that plots for *K* = 6 were still informative with respect to population substructure (Fig. 6a, Additional file 4). In some populations, most individuals were assigned to a single genetic subgroup (Fig. 6a), whereas many individuals showed contributions from multiple subgroups (i.e., admixture). For lineage *B*, STRUCTURE results indicated the presence of two subgroups (Fig. 6a, Additional file 4).

**Fig. 7:**
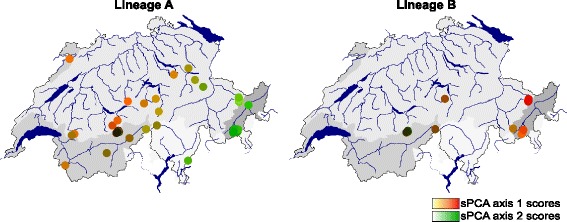
Results of spatial principal component analysis on *B. alpinus* lineages *A* and *B*. Each dot synthesizes the first two sPCA principal components (see [80]), which are plotted on a red (sPCA PC1) or green (sPCA PC2) color scale. The color intensity is proportional to the spatial principal component scores so that populations that are more closely related in multivariate space are similar in color

The within-lineage genetic polymorphisms showed some geographical structure (Figs. 6b and 7). For lineage *A*, an east–west cline in sPCA scores was observed (significant global structure, *P* < 0.001; Fig. 7). The mean population membership probabilities for each of the six genetic subgroups detected within lineage *A* by STRUCTURE (Fig. 6) indicated that different subgroups were common in different regions of the study area: for instance, the "red" subgroup in eastern Switzerland, and "grey" and "pink" in central/western Switzerland (Fig. 6b). Interestingly, there were also several cases where neighbouring sites had substantially different genetic composition. This was especially the case in the centre of the sampling area, where, for instance, a distinct genetic group of lineage *A* was observed in spring-fed tributaries in Loetschental (LT populations; purple in Fig. 6b, dark brown in Fig. 7c).

For lineage *B*, STRUCTURE analyses indicated two genetic subgroups, whereby one subgroup was more abundant in central/western and the other in eastern Switzerland (Fig. 6b, Additional file 4). Populations from the same region (i.e., eastern *vs*. western) tended to be similar even when they were in different major drainages (e.g., Rhine and Danube in eastern Switzerland; Rhine and Rhone in western Switzerland; Fig. 6b). Consistent with the Bayesian clustering results, an east to west gradient in sPCA scores was also evident in lineage *B* (significant global structure, *P* <0.001; Fig. 7). Interestingly, nuclear *B* subgroups matched the mtDNA COI groupings (Fig. 3).

Within lineage population pairwise *F*_ST_s ranged from 0.007 to 0.156 and from 0.003 to 0.142 for lineage *A* and *B*, respectively (Fig. 6c). In both lineages*,* partial Mantel tests indicated significant correlations between pairwise population genetic distances (*F*_ST_) and Euclidean geographical distances once the effect of genetic substructure on population differentiation was taken into account (*A*: *r = 0.135*, *P* = 0.028; *B*: *r = 0.492*, *P* <0.001).

### Morphological differences between *B. alpinus* lineages

The morphological analysis revealed that some morphological traits previously assumed to reflect within species variation [88] were able to consistently distinguish lineages *A* and *B*. The number of setae at the apex of the maxillary palp was higher in *A* (12–18) than in *B* (5–10) as was the number of rows of setae on the dorsal margin of the femora (*A*: 2–3; *B*: 1; see Additional file 6). Moreover, within part of the nuclear subgroups, the triangular spines of the distal margins of tergum IV were more irregular. The margins of the gills IV also presented variation in serration and in the way the setae are grouped. Other characters exhibited variation, but variation in these characters could not be attributed consistently to nuclear subgroups.

## Discussion

### Evidence for cryptic species

In this comprehensive study, we show that the nominal species *B. alpinus* comprises at least two evolutionarily distinct lineages in the central Alps that can be considered separate species under the unified species concept [28]. Several lines of evidence support this interpretation. First, both nuclear and mitochondrial markers clearly separate these two genetic entities and delineate the same genetic groupings, suggesting that these cryptic lineages have been evolving separately for extended periods of time. Second, divergence based on both the between-lineage pairwise *F*_ST_s of microsatellite markers (0.189) and mitochondrial COI sequence divergence (18.9 %) are in agreement with values reported between other congeneric species (e.g., *B. nubecularis* and *B. melanonyx* in our study, see also [19–21, 31]). Third, and most importantly, in spite of widespread sympatric occurrence of both *B. alpinus* lineages in the study area, only 3 % of the 1591 individuals represented intermediate genotypes (i.e., genetically admixed individuals), suggesting at most very rare hybridization. Extremely low levels of gene flow among the lineages are further supported by the observation that individuals consistently clustered by genetic group independent of their geographic origin. For example, individuals from lineage *A* always clustered with other *A* individuals even from geographically distant sites rather than *B* individuals from the same site. Finally, a preliminary morphological analysis of the cryptic lineages, revealed subtle but consistent morphological differences in several traits. In most cases, the observed differences were previously already known but were considered as intraspecific variation [88, 60].

### Biogeography and evolutionary history of the cryptic lineages

Our mtDNA sequence data allowed us to relate genetic diversity of *B. alpinus* from our study region to genetic diversity found in other European studies. We found that *B. alpinus* haplotypes sharing high sequence similarity to those found in Switzerland (our study) have been reported from other European mountain ranges: central Apennines, Ligurian Apennines, Pyrenees and Sierra Nevada (Fig. 3, Additional file 1: Table S3), indicating that both cryptic lineages have a wide European distribution. Furthermore, lineage *A* and *B* individuals from our study showed strong genetic affinities with two of the four unique evolutionary entities previously identified from the Pyrenees and Sierra Nevada [23]. While two lineage *A* haplotypes observed in our study were also found in the Maritime Alps (Fig. 3), there is also clear evidence of genetic differentiation (within each of the cryptic lineages) between samples collected in the central Alps in Switzerland and other European mountain ranges (Fig. 3). This genetic divergence is most likely due to spatial isolation among mountain massifs.

The broad European distribution of the cryptic lineages suggests that they diverged during past geographic isolation (i.e., in allopatry) and were brought into secondary contact upon range expansion. This scenario is further supported by the observation that individuals consistently clustered by genetic lineage rather than by geographic origin, suggesting a common evolutionary history of all populations within a species. Hence, the divergence observed here likely follows a pattern seen in many European plants and animals due to past changes in global climate and related range contractions (and divergence in isolation) and expansions (secondary contact) (see [89] for a review). However, further phylogeographic studies across the entire European distribution range of *B. alpinus* are clearly needed to understand the evolutionary history of these cryptic lineages.

While it seems likely (based on genetic data and known glacial history of Europe) that the initial divergence occurred in allopatry, it is possible that barriers to gene flow between these incipient species have continued to accumulate after secondary contact [90]. Today, multiple types of ecological and non-ecological barriers may contribute to the observed reproductive isolation between the two cryptic lineages. Putative pre-mating barriers could involve, for example, assortative mating based on non-visual cues (e.g., pheromones) or mismatches in genitalia that prevent interspecific mating [91] – as seen in many insect taxa. Premating isolation could also occur if adults from different lineages do not meet during the mating season, for instance, due to differences in the location or time where mating swarms are formed. Spatial and temporal segregation of mating swarms have been reported in several aquatic insects (e.g., [92]), and our own data suggest pronounced phenological differences between lineage *A* and *B* (Leys et al., unpublished data; see also below). Finally, post-zygotic intrinsic barriers due to genetic incompatibilities, as well as extrinsic barriers due to reduced viability or fertility of migrants and hybrids, may play an important role in impeding gene flow and recombination [93]. However, as little is known of mating barriers in baetid mayflies, an important next step is to understand the mechanisms of reproductive isolation in this putative cryptic species complex.

### Differences between the cryptic lineages

The determinants of phenotypically similar but genetically distinct cryptic lineages are diverse, ranging from phenotypic plasticity to non-visual mating signals and morphological stasis [2]. In our study, much of these are still unknown, but analyses of spatial patterns of habitat affiliation, abundance and population genetic variation certainly indicate differences in ecology or population demography – despite relative morphological similarity.

First, the wide geographic ranges and, in particular, frequent sympatric co-occurrence suggest that these *B. alpinus* cryptic lineages can coexist, as also suggested by the patterns observed in other European mountain massifs (e.g., in the Cirque de Taillon in the Pyrenees [23]). Stable coexistence of species is typically thought to require some ecological differentiation or spatio-temporal niche partitioning to reduce inter-specific competition [94]. Our data provide tentative evidence for differences between the lineages in relation to spatial variation in ecological factors (in particular, altitude and stream type): the relative abundance of lineage *B* was greater at high elevations and in spring-fed tributaries. Microhabitat affiliation may result from inter-specific differences in local habitat use by adults for breeding and/or suitable habitats for development of embryos and larvae. Temporal niche segregation may also be important in *B. alpinus*. Our spatio-temporally replicated data in two basins (Leys et al., unpubl. data) indicates that lineage *A* and *B* differ in larval cohort structure and hence in life-history strategies (i.e., voltinism). Ongoing work is investigating to what extent these differences are driven by environmental variation and relate to temporal differences in adult emergence times.

Second, we found that lineage *A* was clearly more abundant than lineage *B* and showed higher levels of population genetic variation. *B. alpinus* lineage *B* appears to be more patchily distributed but relatively more abundant at high elevations, and exhibits lower genetic diversity (in terms of allelic richness and heterozygosity), than lineage *A*. The differences in genetic diversity between lineage *A* and *B* may be explained by spatial distribution and demographic processes*.* A potentially lower effective and demographic population size, lower evolutionary potential and greater extinction risk may be of particular concern with respect to biodiversity loss expected as a result of ongoing global change [95, 7, 10]. In this context, the fate of alpine taxa under climate change may be grim. In particular for taxa, such as the spatially more isolated lineage *B* that appears to have narrower habitat preferences, an upstream distributional shift to higher elevation sites is not possible once glaciers disappear.

### Within lineage substructure

We found clear genetic structure within each of the lineages. In particular, within lineage *A* there are additional genetic subgroups indicating some level of reproductive isolation or reduced gene flow. This possibility is supported by at least two lines of evidence. First, in several cases neighbouring lineage *A* populations showed pronounced differences in genetic composition. A particularly striking example is that the individuals from spring-fed tributaries in Loetschental appear to belong to a genetic cluster completely absent from other nearby sites (purple in Fig. 6a). Moreover, the genetic differentiation between populations of lineage *A*, which are assigned to different genetic subgroups, was largely independent of the geographical distance between them (light grey points labelled "diff" in Fig. 6c). It remains to be further investigated if some of these subgroups result from genetic drift following colonization-extinction dynamics [96, 97] or represent additional evolutionarily distinct units, perhaps adapted to different environmental conditions [98].

We also found clear evidence for east–west genetic clines in both lineages, this pattern being more pronounced in lineage *B* (Figs. 6b and 7). Indeed, the latter lineage showed consistently both nuclear and mtDNA eastern-western spatial structures. These genetic clines do not coincide with any obvious environmental gradient across the study area and could have resulted from past demographic processes during past climatic events (e.g., isolation in allopatry and recolonization dynamics [99]). However, this hypothesis must be further tested using samples across *B. alpinus’* entire distribution range.

## Conclusions

We show that the common *B. alpinus* mayfly comprises at least two distinct evolutionary units within the central Alps. Although these lineages were previously assigned to the same morphospecies based on taxonomic characteristics, our data clearly show that they are genetically, ecologically and morphologically distinct. Our study further emphasizes the need of a phylogenetic reconstruction together with morphological and taxonomic description across the full distributional range of *B. alpinus* to infer the evolutionary history of the cryptic species*.* In general, our study highlights the need to apply molecular analyses of divergence even in taxonomically and ecologically well-studied species: the correct identification of cryptic species is critical for meaningful inferences about biodiversity loss and evolutionary potential in face of global change.

## Availability of supporting data

Nuclear microsatellite data is available in DRYAD (doi: 10.5061/dryad.74458). Mitochondrial COI sequences have been deposited at the ENA database (accessions LT546400 - LT546427). The supporting results and data are provided as Additional files 1, 2, 3, 4, 5 and 6.

## Additional files

Additional file 1: Supplementary tables.
**Table S1.** Sampling site information and population genetic diversity estimates. **Table S2.** Characteristics and amplification conditions of *Baetis alpinus* microsatellite loci used in this study. **Table S3.** List of available published sequences from GenBank and unpublished sequences used in this study. **Table S4.** COI sequence divergence between clades. **Table S5.** Locus-specific and multilocus genetic diversity and differentiation estimates. **Table S6.** Null allele frequency estimates. **Table S7.** Generalized linear model results. (PDF 175 kb) Additional file 2: Bayesian clustering results of STRUCTURE and evidence of multimodality. (PDF 223 kb) Additional file 3: Parsimony haplotype network analysis. (PDF 159 kb) Additional file 4: Results from Bayesian clustering (STRUCTURE). (PDF 898 kb) Additional file 5: Genetic transitive graphs. (PDF 1217 kb) Additional file 6: Morphological analysis of *B. alpinus* lineage larval structures. (PDF 8912 kb)
