# Supplementary material for: Distribution and population genetic variation of cryptic species of the Alpine mayfly Baetis alpinus (Ephemeroptera: Baetidae) in the Central Alps
Source: BMC Evol Biol. 2016 Apr 12;16:77. doi: 10.1186/s12862-016-0643-y (PMC4828801; doi:10.1186/s12862-016-0643-y)
Supplement: Additional file 1: Supplementary tables. — Table S1. Sampling site information and population genetic diversity estimates. Table S2. Characteristics and amplification conditions of Baetis alpinus microsatellite loci used in this study. Table S3. List of available published sequences from GenBank and unpublished sequences used in this study. Table S4. COI sequence divergence between clades. Table S5. Locus-specific and multilocus genetic diversity and differentiation estimates. Table S6. Null allele frequency estimates. Table S7. Generalized linear model results. (PDF 175 kb) [file 12862_2016_643_MOESM1_ESM.pdf]

Additional file 1. Supplementary tables.

Table S1. Sampling site information and population genetic diversity estimates. Sampling site label, scale of the study, main drainage, river, spatial attributes, sample sizes and summary of genetic diversity at 10 gDNA and cDNA microsatellite loci for two *B. alpinus* cryptic species (labelled *A* and *B*) and *B. melanonyx* identified based on Bayesian and ordination clustering methods (see material and methods in main text). L, local; R, regional; N_T, total sample size; *N*, sample size (values below 8 are on grey background); H_E, gene diversity within populations; A_r, allelic richness; A_rP, private allelic richness; A_m mean number of alleles; and F_{IS}, the intrapopulation fixation index (*P <0.0001) are indicated for the two cryptic species. Both A_r and A_rP are based on a minimal sample size of n = 8 diploid individuals using El Mousadik & Petit (1996) and Szpiech *et al.* (2008) rarefaction methods, respectively. Rivers lacking a specific name are indicated as -.

					Spatial coordinates				***B. alpinus* (*A*)**						***B. alpinus* (*B*)**						***B. melanonyx***					
Label	Study	Drainage	River	Locality	Longitude	Latitude	Elevation (m.a.s.l)	N_T	*N*	H_E	A_r	A_rP	A_m	F_{IS}	*N*	H_E	A_r	A_rP	A_m	F_{IS}	*N*	H_E	A_r	A_rP	A_m	F_{IS}
RM1	L	Danube	Ova da Roseg	Val Roseg	9.85867	46.41064	2106	19	16	0.76	6.355	0.209	8.1	0.160*	3	-	-	-	-	-	0	-	-	-	-	-
RM2	L	Danube	Ova da Roseg	Val Roseg	9.85753	46.41831	2054	35	10	0.722	5.989	0.163	6.6	0.127	25	0.571	4.099	0.124	5.6	0.033	0	-	-	-	-	-
RM3	L	Danube	Ova da Roseg	Val Roseg	9.88119	46.45869	1907	34	29	0.771	6.728	0.300	10.9	0.073	5	-	-	-	-	-	0	-	-	-	-	-
RT1	L	Danube	Ova da Roseg	Val Roseg	9.84142	46.39636	2238	38	1	-	-	-	-	-	37	0.530	3.766	0.1	6	0.052	0	-	-	-	-	-
RT2	L	Danube	Ova da Roseg	Val Roseg	9.85636	46.42133	2053	43	4	-	-	-	-	-	39	0.541	3.803	0.102	6	0.033	0	-	-	-	-	-
RT3	L	Danube	Ova da Roseg	Val Roseg	9.86381	46.42564	2017	40	12	0.741	6.119	0.209	7.2	-0.001	28	0.551	3.993	0.136	6.1	0.015	0	-	-	-	-	-
RT4	L	Danube	Ova da Roseg	Val Roseg	9.87458	46.44594	1964	32	4	-	-	-	-	-	28	0.566	3.856	0.314	5.3	0.047	0	-	-	-	-	-
JM1	L	Rhine	Jöribach	Jörital	9.97392	46.78758	2283	40	10	0.806	6.833	0.330	7.6	0.032	30	0.621	5.027	0.45	8	0.072	0	-	-	-	-	-
JM2	L	Rhine	Jöribach	Jörital	9.97286	46.82083	1880	40	34	0.802	6.586	0.175	11.7	0.057	6	-	-	-	-	-	0	-	-	-	-	-
JT1	L	Rhine	Jöribach	Jörital	9.97231	46.77767	2470	20	10	0.803	6.623	0.029	7.2	0.053	10	0.621	4.61	0.273	5.0	0.05	0	-	-	-	-	-
JT2	L	Rhine	Jöribach	Jörital	9.97508	46.78299	2403	40	6	-	-	-	-	-	34	0.595	4.463	0.261	7.2	0.036	0	-	-	-	-	-
JT3	L	Rhine	Jöribach	Jörital	9.98033	46.79267	2198	40	8	0.784	5.700	0.164	5.7	0.059	32	0.609	4.656	0.376	7.8	0.014	0	-	-	-	-	-
JT4	L	Rhine	Jöribach	Jörital	9.981	46.8065	1971	40	5	-	-	-	-	-	35	0.606	4.757	0.254	7.6	0.06	0	-	-	-	-	-
JT5	L	Rhine	Jöribach	Jörital	9.97158	46.82583	1823	40	37	0.773	6.043	0.122	10.5	0.067	3	-	-	-	-	-	0	-	-	-	-	-
LM1	L	Rhone	Anunbach	Jegital	7.89767	46.46106	2217	28	27	0.746	5.37	0.01	7.3	0.037	1	-	-	-	-	-	0	-	-	-	-	-
LM2	L	Rhone	Lonza	Lötschental	7.909	46.44922	2031	22	22	0.756	6.316	0.115	9.3	0.05	0	-	-	-	-	-	0	-	-	-	-	-
LM3	L	Rhone	Lonza	Lötschental	7.89519	46.44436	1954	29	29	0.771	6.606	0.119	10.7	0.047	0	-	-	-	-	-	0	-	-	-	-	-
LM4	L	Rhone	Lonza	Lötschental	7.88553	46.44214	1888	31	30	0.747	6.366	0.171	10.5	0.08	1	-	-	-	-	-	0	-	-	-	-	-

LM5	L	Rhone	Lonza	Lötschental	7.86225	46.43386	1770	38	37	0.773	6.646	0.144	10.9	0.074	0	-	-	-	-	-	1	-	-	-	-	-
LT1	L	Rhone	Anunbach	Jegital	7.89825	46.46031	2215	40	32	0.76	5.591	0.050	8.1	0.104	8	0.396	2.800	0.039	2.8	0.305	0	-	-	-	-	-
LT2	L	Rhone	-	Lötschental	7.90175	46.45144	2111	35	8	0.623	-	-	4.3	0.007	27	0.419	2.938	0.054	3.9	0.09	0	-	-	-	-	-
LT3	L	Rhone	-	Lötschental	7.88708	46.44653	2038	37	14	0.675	5.124	0.044	6.2	0.027	21	0.490	3.522	0.19	4.7	0.038	2	-	-	-	-	-
LT4	L	Rhone	-	Lötschental	7.87547	46.44192	1968	41	10	0.676	4.898	0.004	5.3	-0.036	16	0.484	3.398	0.046	4.1	0.044	15	0.546	2.067	0.831	3.8	0.198*
LT5	L	Rhone	-	Lötschental	7.87008	46.43767	1830	39	34	0.634	4.483	0.046	6.8	0.013	0	-	-	-	-	-	5	-	-	-	-	-
Da1	R	Danube	Ova da Morteratsch	Morteratsch	9.93328	46.42556	2032	34	34	0.752	6.327	0.227	10.6	0.077	0	-	-	-	-	-	0	-	-	-	-	-
Da2	R	Danube	Ova da Morteratsch	Morteratsch	9.94125	46.45028	1899	35	35	0.734	5.97	0.122	10.3	0.101	0	-	-	-	-	-	0	-	-	-	-	-
Da3	R	Danube	-	Val Macun	10.13625	46.73056	2434	19	15	0.772	6.107	0.161	7.8	0.093	4	-	-	-	-	-	0	-	-	-	-	-
Po1	R	Po	Ticino	Fontana	8.55642	46.51847	1240	40	39	0.795	6.677	0.203	12.2	0.065	0	-	-	-	-	-	1	-	-	-	-	-
Po2	R	Po	-	Bogno	9.07819	46.09653	1526	28	28	0.748	5.817	0.333	9.3	-0.003	0	-	-	-	-	-	0	-	-	-	-	-
Ri1	R	Rhine	Gelgia	Bivio	9.69936	46.46497	2033	40	3	-	-	-	-	-	35	0.601	4.741	0.315	8.4	0.035	2	-	-	-	-	-
Ri2	R	Rhine	Lavtinabach	Pizol	9.36575	46.96208	1844	40	33	0.764	6.272	0.285	10.5	0.038	7	-	-	-	-	-	0	-	-	-	-	-
Ri3	R	Rhine	Furshbach	Fursh	9.22743	47.04449	1989	23	23	0.749	5.659	0.081	7.1	0.179*	0	-	-	-	-	-	0	-	-	-	-	-
Ri4	R	Rhine	Wängibach	Speer	9.111211	47.185003	1372	45	10	0.782	5.956	0.111	6.4	0.097	1	-	-	-	-	-	34	0.541	2.223	1.083	5.8	0.283*
Ri5	R	Rhine	Chratzerlibach	Chli Aubrig	8.8628	47.11181	1310	12	12	0.779	6.069	0.248	7.1	0.072	0	-	-	-	-	-	0	-	-	-	-	-
Ri6	R	Rhine	Reuss	Wattingen	8.59403	46.68267	1015	39	39	0.764	6.261	0.281	11.5	0.097*	0	-	-	-	-	-	0	-	-	-	-	-
Ri7	R	Rhine	Engelberger Aa	Surenenpass	8.54317	46.83147	2089	40	25	0.779	6.267	0.137	9.6	0.148*	15	0.529	4.046	0.477	5	-0.033	0	-	-	-	-	-
Ri8	R	Rhine	-	Engstlenalp	8.34072	46.77886	1827	31	19	0.782	5.681	0.466	7.1	0.013	4	-	-	-	-	-	8	0.493	1.864	0.688	3.5	0.341*
Ri9	R	Rhine	Waldemme	Sörenberg	8.06475	46.80481	1165	12	12	0.732	5.808	0.113	6.8	0.106	0	-	-	-	-	-	0	-	-	-	-	-
Ri10	R	Rhine	Briggbach	Grosse Sheidegg	8.09286	46.64786	1688	37	3	-	-	-	-	-	0	-	-	-	-	-	34	0.435	2.922	0.583	4.1	0.284*
Ri11	R	Rhine	-	Mürren	7.88272	46.58186	1797	28	27	0.697	4.752	0.028	6.2	0.013	1	-	-	-	-	-	0	-	-	-	-	-
Ri12	R	Rhine	-	Augstchummi	7.79456	46.52219	1863	40	40	0.729	5.487	0.197	8.2	0.174*	0	-	-	-	-	-	0	-	-	-	-	-
Ri13	R	Rhine	Torneresse	L'Etivaz	7.14747	46.42272	1150	20	20	0.836	7.055	0.278	9.9	0.091	0	-	-	-	-	-	0	-	-	-	-	-
Ri14	R	Rhine	L'Hongrin	Les Mosses	7.103556	46.409333	1400	14	13	0.844	7.229	0.399	8.9	0.071	0	-	-	-	-	-	1	-	-	-	-	-
Ro1	R	Rhone	Chilchenwengbach	Nufenenpass	8.36997	46.47628	1900	40	13	0.763	5.689	0.168	6.7	0.043	20	0.534	4.336	0.443	5.9	0.083	7	-	-	-	-	-
Ro2	R	Rhone	Le Rhône	Grengiols	8.08539	46.37322	860	40	38	0.802	6.227	0.12	9.8	0.06	0	-	-	-	-	-	2	-	-	-	-	-
Ro3	R	Rhone	Turtmänna	Meinden	7.69742	46.19728	1860	40	39	0.788	6.000	0.125	9.8	0.034	1	-	-	-	-	-	0	-	-	-	-	-
Ro4	R	Rhone	Le Trient	Trient	6.99417	46.05222	1307	36	36	0.806	6.525	0.357	11.6	0.081	0	-	-	-	-	-	0	-	-	-	-	-
Ro5	R	Rhone	Le Doubs	Les Moulins	7.05886	47.30538	476	17	16	0.749	5.768	0.348	7.7	0.124	1	-	-	-	-	-	0	-	-	-	-	-
Mean locus estimates										0.758	6.049	0.182	8.54	0.070*		0.5449	4.048	0.233	5.8	0.048*		0.504	2.269	0.796	4.3	0.246*

References:

El Mousadik A, Petit RJ: **High level of genetic differentiation for allelic richness among populations of the argan tree *Argania spinosa* (L) Skeels endemic to Morocco**. *Theoretical and Applied Genetics* 1996, **92**(7):832-839

Szpiech ZA, Jakobsson M, Rosenberg NA: **ADZE: a rarefaction approach for counting alleles private to combinations of populations**. *Bioinformatics* 2008, **24**(21):2498-2504

Table S2. Characteristics and amplification conditions of *Baetis alpinus* microsatellite loci used in this study. Observed numbers of alleles and allelic size ranges are provided in Table S4.

Name	GenBank Accession n°		Primer sequence (5'-3')	Repeat type	Primer concentration (uM)[a]	Dye
**Multiplex set 1**						
Ba_g1		F :	TGCATCGAGGGACACAATCG	$(AC)_{12}$	0.04	FAM
		R :	*TAAAGAGAAAAACTGGTCCTCGC			
Ba_g2		F :	TGAGCAGATTACCACCACCG	$(GT)_{14}$	0.04	FAM
		R :	GCGATGGGTTCGGAAATGAG			
Ba_g3		F :	GACGACTAGTGAGAGCAGGC	$(TG)_{12}$	0.04	ATTO565
		R :	*ACGCGGCCCAATTATCTCTC			
Ba_g4		F :	TTGAGCATCGCCGAATCAAC	$(GA)_{13}$	0.06	ATTO550
		R :	TTCGCTCTGGTGTATGGGTG			
Ba_c1		F :	CTGCGCACTAAACCCTTCTG	$(CA)_{12}$	0.04	ATTO532
		R :	*TCTAGCGGTTAAAGTGTTCTCG			
**Multiplex set 2**						
Ba_g5		F :	TGTTCGTCTCTATCCCGCAG	$(AC)_{17}$	0.08	FAM
		R :	*TGTAACTCTCTCGGTGATGTC			
Ba_g6		F :	GCGCGTTTTTAACTCAAATGAAAG	$(TG)_{16}$	0.08	FAM
		R :	*TAGGCAGGCAAGGCGGAC			
Ba_c2		F :	TCAGGAAACCACTTCCCGAG	$(GCG)_{7}$	0.04	ATTO565
		R :	GCATGTCCACGCCATGTATC			
Ba_c3		F :	TATCCAATCCCACGCACTCC	$(AC)_{11}$	0.04	ATTO550
		R :	GAAGCGGACGTGGTTATGTG			

Ba_c4	F :	TGGCTCCTCCAAGTACAACG	$(AC)_{13}$	0.08	ATTO532
	R :	*CGTACGCACACACTGACATC			

[a] Primer concentration indicates the concentration of the respective primers in the multiplex reaction.

* indicates "pigtail" sequence as described in Brownstein *et al*. (1996).

Reference:

Brownstein, MJ., J. D. Carpten, Smith JR: **Modulation of non-templated nucleotide addition by taq DNA polymerase: Primer modifications that facilitate genotyping**. *Biotechniques* 1996, **20**(6): 1004-1010

Table S3. List of available published sequences from GenBank and unpublished sequences used in this study. Species, GenBank accession number, label used in the reference study, haplotype label used in our study, mountain range and country where the samples were collected, and reference study or provider are shown.

Species	Accession	Specimen	Haplotype	Mountain range	Country	Reference
B. alpinus (ss)	LT546400	Ri12-1-1	alp_A_H1	Central Alps	Switzerland	This study
B. alpinus (ss)	LT546400	Da3-1-4	alp_A_H1	Central Alps	Switzerland	This study
B. alpinus (ss)	LT546401	Ri12-1-3	alp_A_H2	Central Alps	Switzerland	This study
B. alpinus (ss)	LT546402	Ri8-1-2	alp_A_H3	Central Alps	Switzerland	This study
B. alpinus (ss)	LT546403	Ri8-1-3	alp_A_H4	Central Alps	Switzerland	This study
B. alpinus (ss)	LT546404	Po1-1-2	alp_A_H5	Central Alps	Switzerland	This study
B. alpinus (ss)	LT546404	Po1-3-2	alp_A_H5	Central Alps	Switzerland	This study
B. alpinus (ss)	LT546405	Po1-4-2	alp_A_H6	Central Alps	Switzerland	This study
B. alpinus (ss)	LT546405	Ro2-1-1	alp_A_H6	Central Alps	Switzerland	This study
B. alpinus (ss)	LT546406	Po1-5-4	alp_A_H7	Central Alps	Switzerland	This study
B. alpinus (ss)	LT546406	Ro2-1-3	alp_A_H7	Central Alps	Switzerland	This study
B. alpinus (ss)	LT546406	LT1-1-1	alp_A_H7	Central Alps	Switzerland	This study
B. alpinus (ss)	LT546406	Da3-1-5	alp_A_H7	Central Alps	Switzerland	This study
B. alpinus (ss)	LT546406	Da1-1-2	alp_A_H7	Central Alps	Switzerland	This study
B. alpinus (ss)	LT546406	RM3-3-3	alp_A_H7	Central Alps	Switzerland	This study
B. alpinus (ss)	LT546406	RT3-4-1	alp_A_H7	Central Alps	Switzerland	This study
B. alpinus (ss)	LT546407	JT5-1-1	alp_A_H8	Central Alps	Switzerland	This study
B. alpinus (ss)	LT546408	Ri2-1-1	alp_A_H9	Central Alps	Switzerland	This study
B. alpinus (ss)	LT546409	LM1-4-5	alp_A_H10	Central Alps	Switzerland	This study
B. alpinus (ss)	LT546410	LM3-1-1	alp_A_H11	Central Alps	Switzerland	This study
B. alpinus (ss)	LT546411	LM3-1-4	alp_A_H12	Central Alps	Switzerland	This study
B. alpinus (ss)	LT546411	LT1-1-6	alp_A_H12	Central Alps	Switzerland	This study
B. alpinus (ss)	LT546412	LM3-5-4	alp_A_H13	Central Alps	Switzerland	This study
B. alpinus (ss)	LT546413	LT1-1-2	alp_A_H14	Central Alps	Switzerland	This study

B. alpinus (ss)	LT546413	LT5-1-1	alp_A_H14	Central Alps	Switzerland	This study
B. alpinus (ss)	LT546414	LT2-1-2	alp_A_H15	Central Alps	Switzerland	This study
B. alpinus (ss)	LT546415	LT2-1-3	alp_A_H16	Central Alps	Switzerland	This study
B. alpinus (ss)	LT546416	RT3-3-3	alp_A_H17	Central Alps	Switzerland	This study
B. alpinus (ss)	LT546417	Ro3-4-4	alp_A_H18	Central Alps	Switzerland	This study
B. alpinus (ss)	LT546417	Ro3-4-7	alp_A_H18	Central Alps	Switzerland	This study
B. alpinus (ss)	LT546418	Ri1-1-2	alp_Be_H1	Central Alps	Switzerland	This study
B. alpinus (ss)	LT546418	JT5-1-5	alp_Be_H1	Central Alps	Switzerland	This study
B. alpinus (ss)	LT546419	Ri1-1-3	alp_Be_H2	Central Alps	Switzerland	This study
B. alpinus (ss)	LT546420	RM3-1-3	alp_Be_H3	Central Alps	Switzerland	This study
B. alpinus (ss)	LT546420	RT3-1-1	alp_Be_H3	Central Alps	Switzerland	This study
B. alpinus (ss)	LT546421	RM3-1-8	alp_Be_H4	Central Alps	Switzerland	This study
B. alpinus (ss)	LT546422	Ri8-1-1	alp_Bw_H1	Central Alps	Switzerland	This study
B. alpinus (ss)	LT546423	Ri8-2-2	alp_Bw_H2	Central Alps	Switzerland	This study
B. alpinus (ss)	LT546423	LT3-1-2	alp_Bw_H2	Central Alps	Switzerland	This study
B. alpinus (ss)	LT546423	LT3-1-3	alp_Bw_H2	Central Alps	Switzerland	This study
B. alpinus (ss)	LT546424	LT2-1-8	alp_Bw_H3	Central Alps	Switzerland	This study
B.melanonyx	LT546425	Ri8-2-3	mel_CH_H2	Central Alps	Switzerland	This study
B.melanonyx	LT546426	Ri4-1-2	mel_CH_H3	Central Alps	Switzerland	This study
B.melanonyx	LT546427	Ri10-1-1	mel_CH_H4	Central Alps	Switzerland	This study
B. alpinus (ss)	JQ683695	NA	SN_Pyr_H23	Pyrenees	France	Finn et al.(2013) *Global Ecol. Biogeogr.*,22:40-51
B. alpinus (ss)	JQ683694	NA	SN_Pyr_H22	Pyrenees	France	Finn et al.(2013) *Global Ecol. Biogeogr.*,22:40-51
B. alpinus (ss)	JQ683693	NA	SN_Pyr_H21	Pyrenees	France	Finn et al.(2013) *Global Ecol. Biogeogr.*,22:40-51
B. alpinus (ss)	JQ683692	NA	SN_Pyr_H20	Pyrenees	France	Finn et al.(2013) *Global Ecol. Biogeogr.*,22:40-51
B. alpinus (ss)	JQ683691	NA	SN_Pyr_H19	Pyrenees	France	Finn et al.(2013) *Global Ecol. Biogeogr.*,22:40-51
B. alpinus (ss)	JQ683690	NA	SN_Pyr_H18	Pyrenees	France	Finn et al.(2013) *Global Ecol. Biogeogr.*,22:40-51
B. alpinus (ss)	JQ683689	NA	SN_Pyr_H17	Pyrenees	France	Finn et al.(2013) *Global Ecol. Biogeogr.*,22:40-51
B. alpinus (ss)	JQ683688	NA	SN_Pyr_H16	Pyrenees	France	Finn et al.(2013) *Global Ecol. Biogeogr.*,22:40-51
B. alpinus (ss)	JQ683687	NA	SN_Pyr_H15	Pyrenees	France	Finn et al.(2013) *Global Ecol. Biogeogr.*,22:40-51
B. alpinus (ss)	JQ683686	NA	SN_Pyr_H14	Pyrenees	France	Finn et al.(2013) *Global Ecol. Biogeogr.*,22:40-51

B. alpinus (ss)	JQ683685	NA	SN_Pyr_H13	Pyrenees	France	Finn et al.(2013) *Global Ecol. Biogeogr.*,22:40-51
B. alpinus (ss)	JQ683684	NA	SN_Pyr_H12	Pyrenees	France	Finn et al.(2013) *Global Ecol. Biogeogr.*,22:40-51
B. alpinus (ss)	JQ683683	NA	SN_Pyr_H11	Pyrenees	France	Finn et al.(2013) *Global Ecol. Biogeogr.*,22:40-51
B. alpinus (ss)	JQ683682	NA	SN_Pyr_H10	Pyrenees	France	Finn et al.(2013) *Global Ecol. Biogeogr.*,22:40-51
B. alpinus (ss)	JQ683681	NA	SN_Pyr_H9	Pyrenees	France	Finn et al.(2013) *Global Ecol. Biogeogr.*,22:40-51
B. alpinus (ss)	JQ683680	NA	SN_Pyr_H8	Pyrenees	France	Finn et al.(2013) *Global Ecol. Biogeogr.*,22:40-51
B. alpinus (ss)	JQ683679	NA	SN_Pyr_H7	Pyrenees	France	Finn et al.(2013) *Global Ecol. Biogeogr.*,22:40-51
B. alpinus (ss)	JQ683678	NA	SN_Pyr_H6	Pyrenees	France	Finn et al.(2013) *Global Ecol. Biogeogr.*,22:40-51
B. alpinus (ss)	JQ683677	NA	SN_Pyr_H5	Pyrenees	France	Finn et al.(2013) *Global Ecol. Biogeogr.*,22:40-51
B. alpinus (ss)	JQ683676	NA	SN_Pyr_H4	Pyrenees	France	Finn et al.(2013) *Global Ecol. Biogeogr.*,22:40-51
B. alpinus (ss)	JQ683675	NA	SN_Pyr_H3	Pyrenees	France	Finn et al.(2013) *Global Ecol. Biogeogr.*,22:40-51
B. alpinus (ss)	JQ683674	NA	SN_Pyr_H2	Pyrenees	France	Finn et al.(2013) *Global Ecol. Biogeogr.*,22:40-51
B. alpinus (ss)	JQ683673	NA	SN_Pyr_H1	Pyrenees	France	Finn et al.(2013) *Global Ecol. Biogeogr.*,22:40-51
B. alpinus (ss)	KF468683	NA	Picos_A_H1	Picos de Europa	Spain	Finn et al. (2014) *Freshwater Sciences*, 33:1-11
B. alpinus (ss)	KF468682	NA	Pyr_B_H4	Pyrenees	France	Finn et al. (2014) *Freshwater Sciences*, 33:1-11
B. alpinus (ss)	KF468681	NA	Pyr_B_H3	Pyrenees	France	Finn et al. (2014) *Freshwater Sciences*, 33:1-11
B. alpinus (ss)	KF468680	NA	Pyr_B_H2	Pyrenees	France	Finn et al. (2014) *Freshwater Sciences*, 33:1-11
B. alpinus (ss)	KF468679	NA	Pyr_B_H1	Pyrenees	France	Finn et al. (2014) *Freshwater Sciences*, 33:1-11
B. alpinus (ss)	KF468678	NA	SN_B_H5	Sierra Nevada	Spain	Finn et al. (2014) *Freshwater Sciences*, 33:1-11
B. alpinus (ss)	KF468677	NA	SN_B_H4	Sierra Nevada	Spain	Finn et al. (2014) *Freshwater Sciences*, 33:1-11
B. alpinus (ss)	KF468676	NA	SN_B_H3	Sierra Nevada	Spain	Finn et al. (2014) *Freshwater Sciences*, 33:1-11
B. alpinus (ss)	KF468675	NA	SN_B_H2	Sierra Nevada	Spain	Finn et al. (2014) *Freshwater Sciences*, 33:1-11
B. alpinus (ss)	KF468674	NA	SN_B_H1	Sierra Nevada	Spain	Finn et al. (2014) *Freshwater Sciences*, 33:1-11
B. alpinus (ss)	KF468673	NA	SN_Pyr_H25	Sierra Nevada	Spain	Finn et al. (2014) *Freshwater Sciences*, 33:1-11
B. alpinus (ss)	KF468672	NA	SN_Pyr_H24	Sierra Nevada	Spain	Finn et al. (2014) *Freshwater Sciences*, 33:1-11
B. alpinus (sl)	HG935018	BA-47-IT(LI)	BA-47-IT	Ligurian Appenines	Italia	Gattolliat et al. (2015) *Arthropod systematics and Phylogeny*, 73:3-18
B. alpinus (sl)	HG935017	BA-45-IT(LI)	BA-45-IT	Ligurian Appenines	Italia	Gattolliat et al. (2015) *Arthropod systematics and Phylogeny*, 73:3-18
B. alpinus (sl)	HG935016	BA-44-FR(ME)	alp_A_H13	Maritime Alps	France	Gattolliat et al. (2015) *Arthropod systematics and Phylogeny*, 73:3-18
B. alpinus (sl)	HG935015	BA-43-FR(ME)	alp_A_H9	Maritime Alps	France	Gattolliat et al. (2015) *Arthropod systematics and Phylogeny*, 73:3-18
B. alpinus (sl)	HG935013	BA-36-FR(MV)	BA-36-FR	Maritime Alps	France	Gattolliat et al. (2015) *Arthropod systematics and Phylogeny*, 73:3-18

B. alpinus (sl)	HG935012	BA-34-IT(AP)	BA-34-IT	Central Appenines	Italia	Gattolliat et al. (2015) *Arthropod systematics and Phylogeny*, 73:3-18
B. alpinus (sl)	HG935011	BA-33-IT(AP)	BA-33-IT	Central Appenines	Italia	Gattolliat et al. (2015) *Arthropod systematics and Phylogeny*, 73:3-18
B.melanonyx	NA	FR_SR28D05_B	mel_FR_H1	NA	France	Unpublished (S. Rutschmann & M.T. Monaghan)
B.nubecularis	NA	CH041_SR21F05_B	nub_CH_H1	Central Alps	Switzerland	Unpublished (S. Rutschmann & M.T. Monaghan)
B.nubecularis	NA	CH051_SR21H08_B	nub_CH_H2	Central Alps	Switzerland	Unpublished (S. Rutschmann & M.T. Monaghan)
B. rhodani	KF438126	CH_745580_neo	rhodani	Central Alps	Switzerland	Rutschmann et al. (2014) *Freshwater biology*, 59: 2516–2527

References:

Finn DS, Khamis K, Milner AM: **Loss of small glaciers will diminish beta diversity in Pyrenean streams at two levels of biological organization**. *Global Ecology and Biogeography* 2013, **22**(1):40-51.

Finn DS, Zamora-Muñoz C, Múrria C, Sáinz-Bariáin M, Alba-Tercedor J: **Evidence from recently deglaciated mountain ranges that *Baetis alpinus* (Ephemeroptera) could lose significant genetic diversity as alpine glaciers disappear**. *Freshwater Science* 2014, **33**(1):207-216.

Gattolliat J-L, Cavallo E, Vuataz L, Sartori M: **DNA barcoding of Corsican mayflies (Ephemeroptera) with implications on biogeography, systematics and biodiversity**. *Arthropod Systematics & Phylogeny* 2015, **73**(1):3-18

Rutschmann S, Gattolliat J-L, Hughes SJ, Baez M, Sartori M, Monaghan MT: **Evolution and island endemism of morphologically cryptic *Baetis* and *Cloeon* species (Ephemeroptera, Baetidae) on the Canary Islands and Madeira**. *Freshwater Biology* 2014, **59**:2516-2527.

Table S4. COI sequence divergence between clades. Mean % sequence divergence between *Baetis alpinus* clades assuming the best-fit (Tamura-Nei) nucleotide substitution model and measured at the Cytochrome Oxidase I (COI) barcoding marker. (Pyr_B, SN_Pyr, SN_B, Picos_A correspond to Generalized Mixed Yule-Coalescent (GMYC) entities identified in Finn *et al*. (2014). *BA-45-IT* and *BA-36-FR* correspond to single haplotypes described in Gattolliat *et al*. (2015).

			B. alpinus						*Baetis*. gr. *alpinus*	
Entities	*B. nubecularis*	*B. melanonyx*	*LinB*	*LinA*	*SN_Pyr*	*Picos_A*	*Pyr_B*	*SN_B*	*BA-45-IT*	*BA-36-FR*
B. nubecularis	0.005									
B. melanonyx	0.249	0.069								
LinB	0.186	0.246	0.045							
LinA	0.049	0.243	0.189	0.012						
SN_Pyr	0.061	0.253	0.182	0.055	0.023					
Picos_A	0.067	0.248	0.183	0.058	0.054	NA				
Pyr_B	0.191	0.274	0.081	0.201	0.198	0.199	0.002			
SN_B	0.189	0.232	0.197	0.193	0.202	0.210	0.194	0.007		
BA-45-IT	0.254	0.256	0.249	0.252	0.255	0.239	0.235	0.243	NA	
BA-36-FR	0.262	0.274	0.271	0.254	0.257	0.245	0.274	0.257	0.156	NA

References:

Finn DS, Zamora-Muñoz C, Múrria C, Sáinz-Bariáin M, Alba-Tercedor J: **Evidence from recently deglaciated mountain ranges that *Baetis alpinus* (Ephemeroptera) could lose significant genetic diversity as alpine glaciers disappear**. *Freshwater Science* 2014, **33**(1):207-216.

Gattolliat J-L, Cavallo E, Vuataz L, Sartori M: **DNA barcoding of Corsican mayflies (Ephemeroptera) with implications on biogeography, systematics and biodiversity**. *Arthropod Systematics & Phylogeny* 2015, **73**(1):3-18

Table S5. Locus-specific and multilocus genetic diversity and differentiation estimates. Locus-specific and multilocus genetic diversity measures and genetic differentiation (F_{ST}) following Weir & Cockerham (1984) estimated across all populations within two *B. alpinus* lineages (*A* and *B*) and *B. melanonyx*. A_t, total number of alleles; A_r, allelic richness; H_O, observed heterozygosity; H_E, and gene diversity; Genetic differentiation (F_{ST}) following Weir & Cockerham (1984). Significance of genetic differentiation was tested using 10,000 randomizations (*$P < 0.0001$). Bonferroni corrections were applied to the mean *F*-statistic estimate. Allelic size ranges in base pairs (bp) are indicated for each locus.

Locus	A_t	A_r	H_O	H_E	Allelic size range(bp)	F_{ST}
***B. alpinus* (*A*)**						
Ba_c1	19	7.017	0.732	0.767	84 - 103	0.093*
Ba_g4	47	8.282	0.807	0.852	93 - 224	0.031*
Ba_g3	35	6.660	0.751	0.809	74 - 163	0.036*
Ba_g2	45	9.490	0.786	0.819	136 - 219	0.093*
Ba_g1	36	7.118	0.731	0.789	62 - 137	0.066*
Ba_g6	19	4.905	0.643	0.663	87 - 133	0.117*
Ba_g5	29	7.561	0.731	0.811	165 - 219	0.058*
Ba_c4	30	8.607	0.834	0.853	199 - 242	0.055*
Ba_c3	38	7.370	0.737	0.802	181 - 228	0.061*
Ba_c2	12	3.633	0.336	0.422	180 - 202	0.160*
Average	31	7.064	0.709	0.759		0.073*
***B. alpinus* (*B*)**						
Ba_c1	14	4.351	0.518	0.598	86 - 101	0.072*
Ba_g4	25	6.190	0.647	0.731	98 - 198	0.046*

Ba_g3	8	3.356	0.530	0.534	76 - 126	0.185*
Ba_g2	26	4.734	0.583	0.587	135 - 182	0.052*
Ba_g1	16	4.945	0.612	0.629	65 - 100	0.148*
Ba_g6	4	1.000	0.075	0.042	93 - 104	0.538*
Ba_g5	8	1.981	0.202	0.179	171 -192	0.065*
Ba_c4	18	7.823	0.755	0.821	211 - 230	0.053*
Ba_c3	40	8.027	0.601	0.748	173 - 238	0.061*
Ba_c2	11	3.914	0.632	0.675	188 - 200	0.039*
Average	17	4.632	0.516	0.554		0.081*
B. melanonyx						
Ba_c1	4	1.344	0.007	0.014	85 - 095	0.000
Ba_g4	22	7.834	0.758	0.926	94 - 157	0.022*
Ba_g3	5	3.852	0.28	0.468	76 - 086	0.059
Ba_g2	13	4.094	0.517	0.785	143 - 178	0.135*
Ba_g1	13	5.661	0.586	0.721	62 - 102	0.176*
Ba_g6	5	2.433	0.021	0.211	87 - 097	0.205*
Ba_g5	4	2.088	0.35	0.358	171 - 192	0.170*
Ba_c4	12	5.583	0.787	0.656	198 - 224	0.159*
Ba_c3	6	2.961	0.286	0.559	189 - 196	0.134*
Ba_c2	11	5.041	0.569	0.57	172 - 201	0.167*
Average	9.5	4.0891	0.416	0.527		0.127*

Reference:

Weir BS, Cockerham CC: **Estimating *F*-statistics for the analysis of population structure**. *Evolution* 1984, **38**(6):1358-1370

Table S6. Null allele frequency estimates. Null allele frequency estimates per locus and population calculated following Dempster *et al.* (1977) in GENEPOP for populations containing a minimum of eight individuals. High probabilities of null alleles (≥0.2) are highlighted in grey.

B. alpinus (A)

	**Ri12**	**Ri8**	**Ri13**	**Po1**	**Ri3**	**Po2**	**JM1**	**JM2**	**JT1**	**JT3**	**JT5**	**Ri2**	**Ro2**	**LM1**	**LM2**	**LM3**	**LM4**	**LM5**	**LT1**	**LT2**	**LT3**
Ba_c1	0.037	0.091	0.001	0	0.118	0.015	0	0.085	0	0	0.016	0.099	0	0.027	0	0	0	0.085	0	0	0
Ba_g4	0.14	0	0	0.039	0.095	0	0.05	0	0	0	0.031	0	0.051	0	0	0.055	0	0.006	0.043	0	0
Ba_g3	0.26	0.076	0.043	0.017	0	0	0.013	0	0	0.015	0.036	0	0	0.031	0	0	0	0	0	0.015	0
Ba_g2	0.048	0	0	0.027	0.125	0	0	0.013	0	0	0.038	0	0	0.038	0.038	0	0.134	0.067	0	0	0.06
Ba_g1	0.058	0	0.131	0.051	0.104	0	0	0	0.033	0	0.022	0.013	0.024	0	0.012	0.04	0.022	0.067	0.056	0	0.081
Ba_g6	0	0.061	0.022	0.002	0	0.058	0	0	0	0.016	0.07	0	0.026	0	0	0	0	0.008	0.042	0	0
Ba_g5	0.01	0	0.044	0	0.133	0.016	0	0.03	0	0	0	0.091	0.003	0.024	0.038	0.081	0.074	0.039	0.038	0	0
Ba_c4	0	0	0	0.034	0	0	0	0.021	0.102	0	0.015	0.028	0	0	0.083	0.029	0.008	0.03	0.053	0	0
Ba_c3	0.027	0.007	0.035	0.015	0.029	0	0	0.043	0	0	0	0	0.043	0.006	0.107	0.017	0.007	0.037	0.065	0.193	0
Ba_c2	0.085	0.08	0.057	0.043	0	0	0.068	0.025	0	0.247	0	0.041	0.017	0.056	0	0.09	0	0	0.086	0	0

	**LT4**	**LT5**	**Da3**	**Da1**	**Da2**	**Ri14**	**Ro5**	**Ri11**	**Ro1**	**RM1**	**RM2**	**RM3**	**RT3**	**Ri9**	**Ri4**	**Ri7**	**Ro4**	**Ro3**	**Ri5**	**Ri6**
Ba_c1	0	0.011	0.063	0	0	0.025	0	0	0	0	0	0.037	0	0	0	0.09	0	0.033	0	0
Ba_g4	0	0	0	0.032	0.008	0	0	0	0	0.038	0	0.076	0	0	0.111	0.083	0.017	0	0	0.04
Ba_g3	0	0	0	0	0.077	0	0.006	0	0.132	0.049	0.086	0.044	0	0	0	0.065	0.036	0	0	0.013
Ba_g2	0	0.036	0.01	0.007	0.023	0.033	0.133	0	0	0.041	0	0.017	0.016	0	0.081	0.015	0	0.048	0	0.024
Ba_g1	0	0	0.006	0.007	0.007	0.187	0	0.014	0.077	0	0	0.043	0	0.067	0.202	0	0.103	0	0.049	0.078
Ba_g6	0	0.007	0.083	0.087	0.052	0.028	0	0	0.021	0	0	0	0	0	0	0.044	0	0.017	0.005	0.014
Ba_g5	0	0.006	0	0.096	0.093	0	0.013	0.035	0	0.161	0.169	0	0.107	0.14	0	0.08	0	0.048	0	0.021
Ba_c4	0.111	0	0.027	0	0	0	0.127	0	0	0.041	0.081	0	0	0	0	0.023	0	0.021	0.039	0.07
Ba_c3	0	0	0.022	0.082	0.081	0	0	0.024	0	0.051	0.144	0.099	0	0.051	0	0.043	0.052	0.011	0	0.097
Ba_c2	0	0	0.067	0	0.06	0	0.134	0.064	0.005	0.238	0	0	0	0	0	0.114	0.058	0.018	0.142	0.012

B. alpinus (B)

	**Ri1**	**JM1**	**JT1**	**JT2**	**JT3**	**JT4**	**LT1**	**LT2**	**LT3**	**LT4**	**Ro1**	**RM2**	**RT1**	**RT2**	**RT3**	**RT4**	**Ri7**
Ba_c1	0.067	0.032	0	0	0	0.035	0.193	0	0.046	0.018	0.069	0.013	0	0.031	0.056	0.037	0.057
Ba_g4	0	0	0.136	0.047	0.048	0.075	0.061	0.03	0.009	0	0.043	0.101	0.021	0	0	0.034	0.025

Ba_g3	0	0.02	0.052	0.063	0	0.083	NA	0	0.067	0	0.106	0.037	0.055	0.006	0.047	0	0.218
Ba_g2	0.027	0	0.067	0.031	0	0.028	0	0.06	0	0	0.066	0	0	0.075	0	0.041	0
Ba_g1	0	0	0	0.002	0	0	0	0.006	0.033	0	0	0.01	0	0	0	0	0
Ba_g6	NA	NA	NA	NA	NA	NA	NA	NA	NA	NA	NA	NA	NA	NA	NA	NA	NA
Ba_g5	0	0.123	0	0	0	0	NA	NA	0	NA	0	0.017	0	0	0	0	0
Ba_c4	0.023	0.008	0	0	0	0	0.187	0.129	0.117	0	0.065	0	0.033	0	0.042	0	0
Ba_c3	0.056	0.097	0	0.061	0.057	0.154	NA	0.063	0	0	0.1	0	0.064	0.051	0.054	0	0
Ba_c2	0.091	0.067	0	0	0.041	0.009	0.042	0	0	0.114	0	0	0	0	0.048	0.04	0

B. melanonyx

	**Ri8**	**Ri10**	**LT4**	**Ri4**
Ba_c1	NA	NA	NA	0.1
Ba_g4	0.205	0.226	0	0.3
Ba_g3	0	0.144	0.016	0.142
Ba_g2	0.319	0.1	0.302	0.072
Ba_g1	0.083	0	0	0.029
Ba_g6	0.224	0	0.267	0.117
Ba_g5	0	0	0	0.058
Ba_c4	0	0	0	0.017
Ba_c3	0	0.31	0.178	0.201
Ba_c2	0.166	0.056	0	0.303

Reference:

Dempster AP, Laird NM, Rubin DB: **Maximum Likelihood from incomplete data via the EM Algorithm** *Journal of the Royal Statistical Society Series B (Methodological)* 1977, **39**:1-38.

Table S7. Generalized linear model results. Generalized linear models determining the factors explaining the relative abundance of *B. alpinus A* and *B* lineages performed at **(A)** the regional scale and **(B)** the local scale (B). Estimates and significance of the fixed effect β parameters of the best fit models indicated in bold are shown.

				Model selection		
	Effect	β parameter estimate	(S.E.)	Deviance	*F*-value	*P*
**A**.	Intercept	9.182	3.166			
	**Elevation**	-0.004	0.002	373.510	12.867	*0.002*
	Drainage			252.380	0.520	0.673
**B**.	Intercept	0.9891	0.67			
	**Stream-type**	-1.8983	0.65	394.8	9.4228	*0.006*
	**Valley** (*Ro*)	1.4571	0.71	393.51	4.6633	*0.022*
	**Valley** (*Da*)	-0.6958	0.75			

The following statistics are given: estimates and standard errors (S.E.) of the β parameters, Deviance, *F*-value and *P*-value of the likelihood ratio *F*-test. *P*-values ≤0.05 are given in italics.
