# Supplementary material for: Distribution and population genetic variation of cryptic species of the Alpine mayfly Baetis alpinus (Ephemeroptera: Baetidae) in the Central Alps
Source: BMC Evol Biol. 2016 Apr 12;16:77. doi: 10.1186/s12862-016-0643-y (PMC4828801; doi:10.1186/s12862-016-0643-y)
Supplement: Additional file 2: — Bayesian clustering results of STRUCTURE and evidence of multimodality. (PDF 223 kb) [file 12862_2016_643_MOESM2_ESM.pdf]

Additional file 2. Bayesian clustering results of STRUCTURE and evidence of multimodality.

Similarity coefficients between runs (i.e. 30 replicates of the same *K*) (*H*') estimated by CLUMPP were low, indicating significant multimodality (*K*= 3: *H*'= 0.725; from 0.743 to 0.999). **(A)** A Non-metric multidimensional scaling performed on the run pairwise matrix of similarity coefficients (*K* = 3) (transformed to a dissimilarity matrix) was used to visualize the different solutions for the 30 replicates. Three distinct groups of solutions were found among the 30 runs. The first, second and third group of solutions comprised 19, 8 and 3 runs, respectively. **(B)** Bayesian assignment probabilities of *B. alpinus* and *B. melanonyx* individuals into three inferred clusters, averaged across the three groups of solutions. Each individual is represented by a vertical line (x axis) partitioned into *K* colored segments that represent the individual's estimated membership proportion in each of the *K* clusters (y axis). Sampling site labels (letters along the x axis) are identical to Figure 1. **(C)** The average individual cluster assignment probabilities of all replicates within the most frequently observed group of solutions (i.e. 19 runs) was used for interpretation (M. Jakobsson, personal communication). Similarity coefficients between the selected runs was high (*H*'= 0.999). **(D)** DAPC cluster assignment probabilities of each individual for the three clusters based on the discriminant functions are shown for comparison.

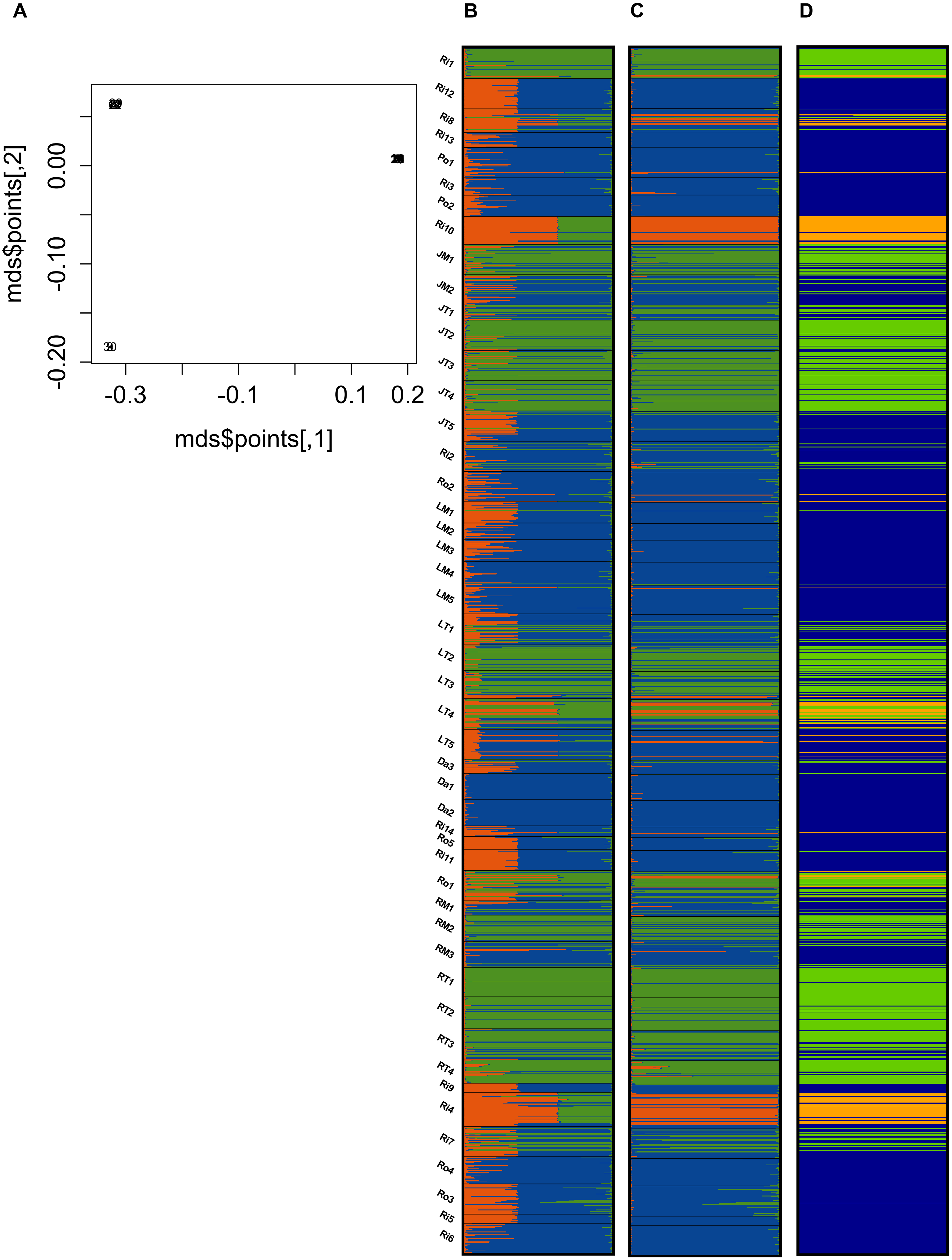
A
B
C
D
mds$points[,2]
0.00
-0.10
-0.20
-0.3
-0.1
0.1
0.2
mds$points[,1]
Ri1
Ri12
Ri8
Ri13
Po1
Ri3
Po2
Ri10
JM1
JM2
JT1
JT2
JT3
JT4
JT5
Ri2
Ro2
LM1
LM2
LM3
LM4
LM5
LT1
LT2
LT3
LT4
LT5
Da3
Da1
Da2
Ri14
Ro5
Ri11
Ro1
RM1
RM2
RM3
RT1
RT2
RT3
RT4
Ri9
Ri4
Ri7
Ro4
Ro3
Ri5
Ri6
