# Supplementary material for: Distribution and population genetic variation of cryptic species of the Alpine mayfly Baetis alpinus (Ephemeroptera: Baetidae) in the Central Alps
Source: BMC Evol Biol. 2016 Apr 12;16:77. doi: 10.1186/s12862-016-0643-y (PMC4828801; doi:10.1186/s12862-016-0643-y)
Supplement: Additional file 3: — Parsimony haplotype network analysis. (PDF 159 kb) [file 12862_2016_643_MOESM3_ESM.pdf]

Additional file 3. Parsimony haplotype network analysis.

Methods: We performed a Parsimony haplotype network analysis using TCS 1.2 (Clement *et al*. 2000). We tested for species and cryptic lineage delineation by asking whether the COI haplotype data lead to multiple separate networks with the 95% plausible connection limit. Haplotypes separated by more mutational steps (i.e., the probability of secondary mutations exceeds 5%) remain disconnected. This approach does not rely on assumptions of phylogenetic reconstruction methods such as bifurcating relationships and recent analyses show that the 95% parsimony connection limit can provide an additional and simple quantitative standard for phylogenetic species (Monaghan *et al*. 2006; Hart & Sunday 2007).

Results: Statistical parsimony analysis of these 70 unique haplotypes revealed the presence of 16 distinct networks (L_{95}=10 steps). In our data, *B. melanonyx* consisted of one haplotype network, and both *B. alpinus* lineage *B* (matching the nuclear subgroup level divergence, see Figure 5) and lineage *A* of two further networks. Of the latter, one network included only two *A* haplotypes. Networks matched all Finn *et al*. GMYC entities (2013;2014) but one, SN_Pyr, split among three networks. Additionally, one network included both *B. nubecularis* haplotypes.

References:

Clement M, Posada D, Crandall KA: **TCS: a computer program to estimate gene genealogies**. *Molecular Ecology* 2000, **9**(10):1657-1659.

Finn DS, Khamis K, Milner AM: **Loss of small glaciers will diminish beta diversity in Pyrenean streams at two levels of biological organization**. *Global Ecology and Biogeography* 2013, **22**(1):40-51.

Finn DS, Zamora-Muñoz C, Múrria C, Sáinz-Bariáin M, Alba-Tercedor J: **Evidence from recently deglaciated mountain ranges that *Baetis alpinus* (Ephemeroptera) could lose significant genetic diversity as alpine glaciers disappear**. *Freshwater Science* 2014, **33**(1):207-216.

Hart MW, Sunday J: **Things fall apart: biological species form unconnected parsimony networks**. *Biology Letters* 2007, **3**(5):509-512

Monaghan MT, Balke M, Pons J, Vogler AP: **Beyond barcodes: complex DNA taxonomy of a south pacific island radiation**. *Proceedings of the Royal Society B-Biological Sciences* 2006, **273**(1588):887-893.

Figure: Network analysis based on statistical parsimony (Clement *et al*. 2000) showing the genealogical relationships of the COI haplotypes in cryptic lineages of *B. alpinus, B. melanonyx* and *B. nubecularis*. The labels in the circles refer to the COI haplotypes (see Figure 3; Additional file 1, Table S3). The empty dots indicate the presence of missing haplotype intermediates while the connections are based on the set of plausible solutions with a 95% of parsimony probability.

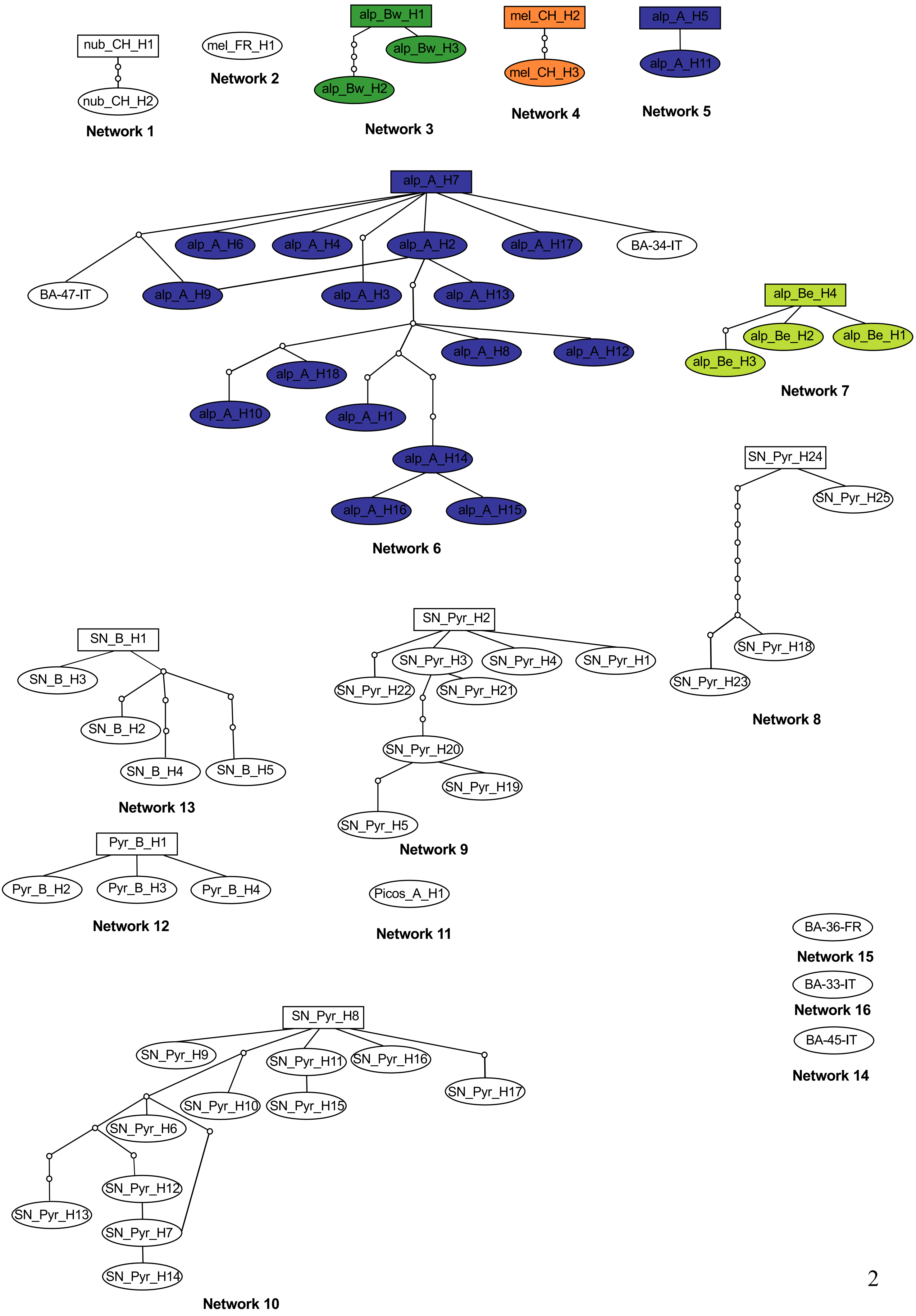
nub_CH_H1
nub_CH_H2
Network 1
mel_FR_H1
Network 2
alp_Bw_H1
alp_Bw_H3
alp_Bw_H2
Network 3
mel_CH_H2
mel_CH_H3
Network 4
alp_A_H5
alp_A_H11
Network 5
alp_A_H7
alp_A_H6
alp_A_H4
alp_A_H2
alp_A_H17
BA-34-IT
BA-47-IT
alp_A_H9
alp_A_H3
alp_A_H13
alp_A_H8
alp_A_H12
alp_A_H18
alp_A_H10
alp_A_H1
alp_A_H14
alp_A_H16
alp_A_H15
Network 6
alp_Be_H4
alp_Be_H2
alp_Be_H1
alp_Be_H3
Network 7
SN_Pyr_H24
SN_Pyr_H25
SN_Pyr_H18
SN_Pyr_H23
Network 8
SN_Pyr_H2
SN_Pyr_H3
SN_Pyr_H4
SN_Pyr_H1
SN_Pyr_H22
SN_Pyr_H21
SN_Pyr_H20
SN_Pyr_H19
SN_Pyr_H5
Network 9
SN_B_H1
SN_B_H3
SN_B_H2
SN_B_H4
SN_B_H5
Network 13
Pyr_B_H1
Pyr_B_H2
Pyr_B_H3
Pyr_B_H4
Network 12
Picos_A_H1
Network 11
BA-36-FR
Network 15
BA-33-IT
Network 16
BA-45-IT
Network 14
SN_Pyr_H8
SN_Pyr_H9
SN_Pyr_H11
SN_Pyr_H16
SN_Pyr_H17
SN_Pyr_H10
SN_Pyr_H15
SN_Pyr_H6
SN_Pyr_H12
SN_Pyr_H13
SN_Pyr_H7
SN_Pyr_H14
Network 10
