# Supplementary material for: Distribution and population genetic variation of cryptic species of the Alpine mayfly Baetis alpinus (Ephemeroptera: Baetidae) in the Central Alps
Source: BMC Evol Biol. 2016 Apr 12;16:77. doi: 10.1186/s12862-016-0643-y (PMC4828801; doi:10.1186/s12862-016-0643-y)
Supplement: Additional file 4: — Results from Bayesian clustering (STRUCTURE). (PDF 898 kb) [file 12862_2016_643_MOESM4_ESM.pdf]

Additional file 4. **Results from Bayesian clustering (STRUCTURE).**

Individual assignment results from Bayesian clustering following Pritchard *et al*. (2000) performed on **(a)** 1591 individuals of the *B. alpinus* group (*s.l.*), including *B. alpinus* and *B. melanonyx* (corrected for multimodality) and, **(b)** *B. alpinus* cryptic lineages and *B. melanonyx* datasets separately. **(c)** STRUCTURE results for separate *B. alpinus* cryptic lineages and *B. melanonyx* datasets: mean (±SD) probabilities of the data Ln Pr(X|*K*) over 30 replicated runs plotted as a function of the putative number of clusters *K* (blue dots) and the standardized second-order rate of change of Ln Pr(X|*K*), *ΔK*, as a function of *K* (red dots).

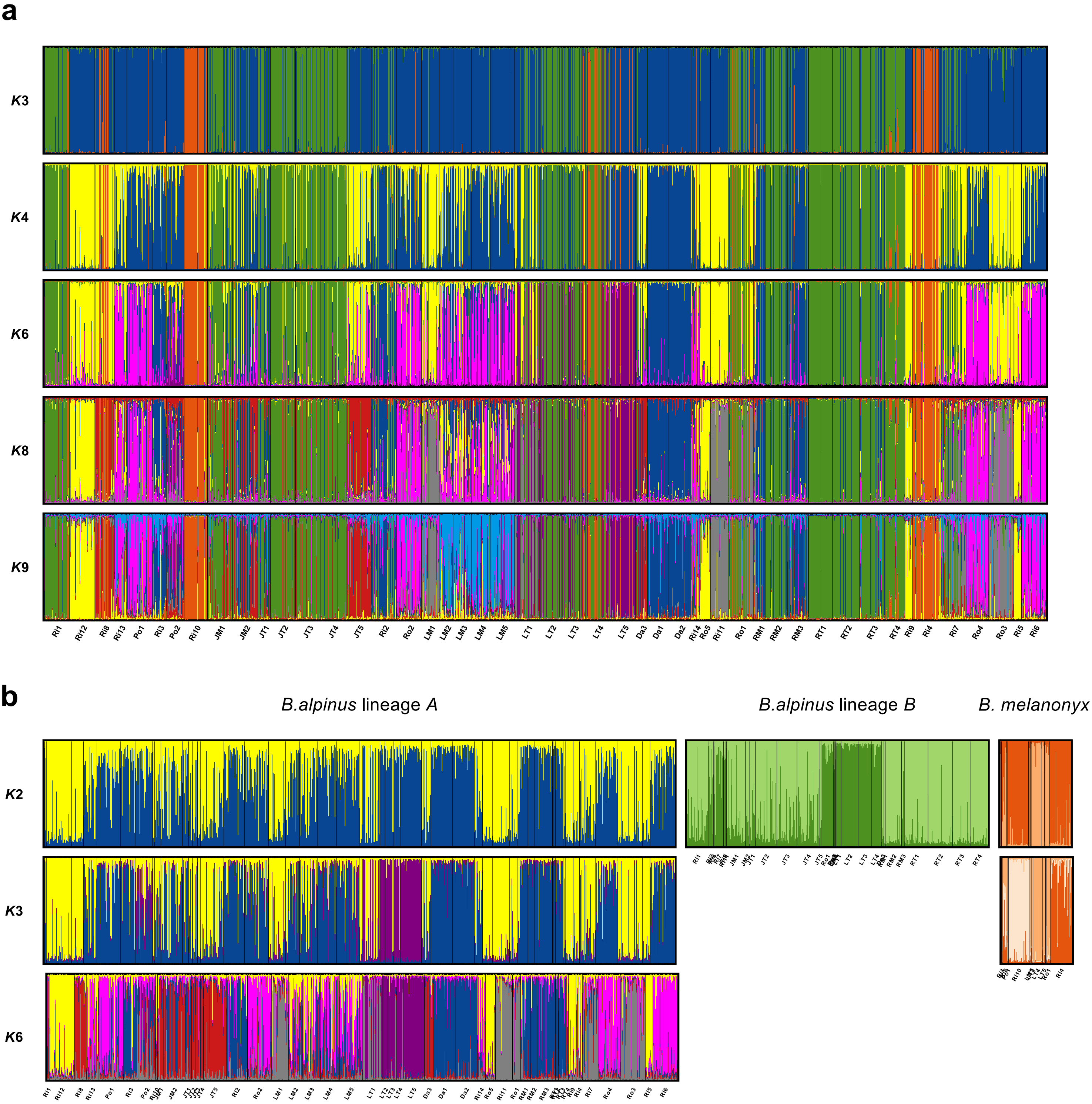

c

B. alpinus* lineage *A

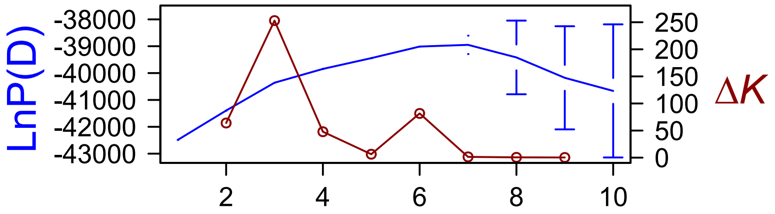

B. alpinus* lineage *B

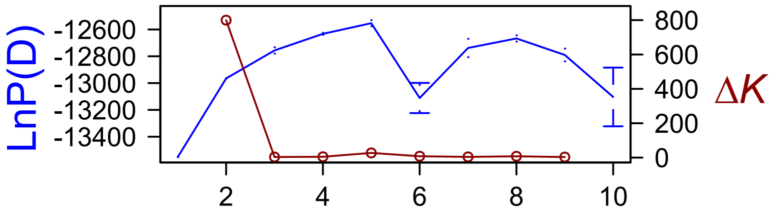

B. melanonyx

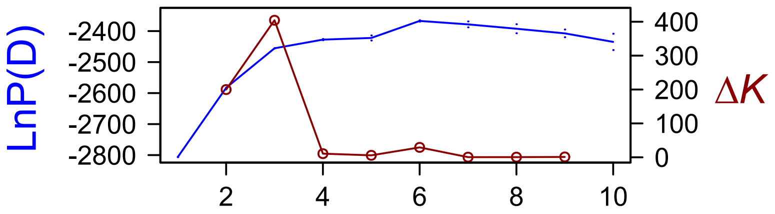

Reference:

Pritchard JK, Stephens M, Donnelly P: **Inference of population structure using multilocus genotype data**. *Genetics* 2000, **155**(2):945-959.
