# Supplementary material for: Distribution and population genetic variation of cryptic species of the Alpine mayfly Baetis alpinus (Ephemeroptera: Baetidae) in the Central Alps
Source: BMC Evol Biol. 2016 Apr 12;16:77. doi: 10.1186/s12862-016-0643-y (PMC4828801; doi:10.1186/s12862-016-0643-y)
Supplement: Additional file 5: — Genetic transitive graphs. (PDF 1217 kb) [file 12862_2016_643_MOESM5_ESM.pdf]

Additional file 5. **Genetic transitive graphs.**

Graph connecting closely related mtDNA COI haplotypes. Clusters were identified using a graph approach where COI haplotype pairs are connected when being less genetically distant than a given threshold distance (Hamming distance) using the function '*gengraph*' of the R package *adegenet* (Jombart *et al*. 2008). The Hamming distance cutoff is shown in the inset. Hamming distances between pairs of connected haplotypes are indicated by the numbers annotating the transitive graph edges. Colors indicate nuclear cluster membership for individuals sequenced in our study. The graph datasets labeled with a number (I and II) were subsequently analyzed with further divergence level using the same approach.

Reference:

Jombart T: **ADEGENET: a R package for the multivariate analysis of genetic markers**. *Bioinformatics* 2008, **24**(11):1403-1405.

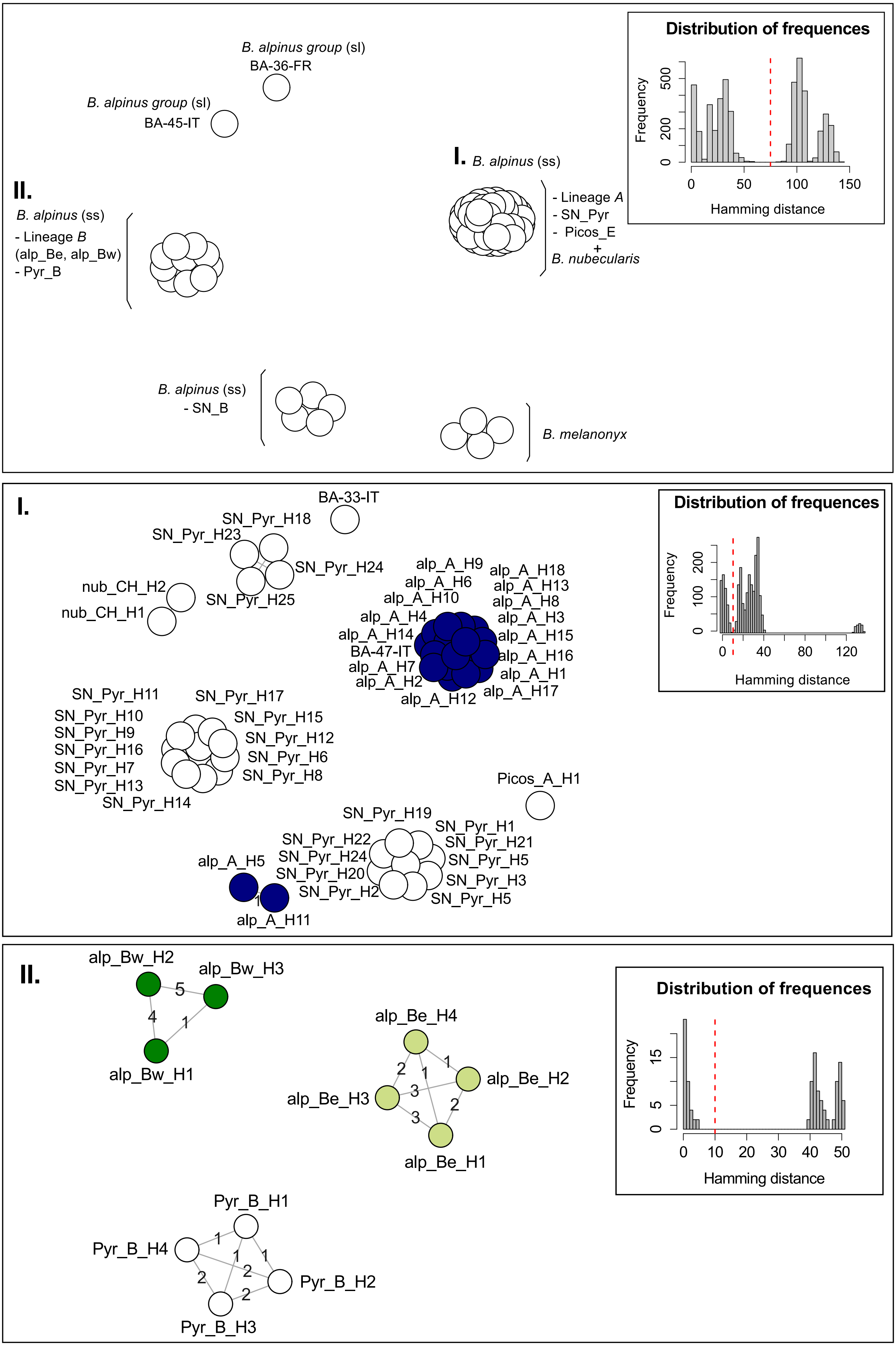
Distribution of frequences
Frequency
0
200
500
0
50
100
150
Hamming distance
B. alpinus group (sl)
BA-36-FR
B. alpinus group (sl)
BA-45-IT
I. B. alpinus (ss)
- Lineage A
- SN_Pyr
- Picos_E
+
B. nubecularis
II.
B. alpinus (ss)
- Lineage B
(alp_Be, alp_Bw)
- Pyr_B
B. alpinus (ss)
- SN_B
B. melanonyx
I.
BA-33-IT
SN_Pyr_H18
SN_Pyr_H23
SN_Pyr_H24
SN_Pyr_H25
nub_CH_H2
nub_CH_H1
alp_A_H9
alp_A_H6
alp_A_H10
alp_A_H4
alp_A_H14
BA-47-IT
alp_A_H7
alp_A_H2
alp_A_H12
alp_A_H18
alp_A_H13
alp_A_H8
alp_A_H3
alp_A_H15
alp_A_H16
alp_A_H1
alp_A_H17
Distribution of frequences
Frequency
0
100
200
0
40
80
120
Hamming distance
SN_Pyr_H11
SN_Pyr_H10
SN_Pyr_H9
SN_Pyr_H16
SN_Pyr_H7
SN_Pyr_H13
SN_Pyr_H14
SN_Pyr_H17
SN_Pyr_H15
SN_Pyr_H12
SN_Pyr_H6
SN_Pyr_H8
Picos_A_H1
SN_Pyr_H19
SN_Pyr_H22
SN_Pyr_H24
SN_Pyr_H20
SN_Pyr_H2
SN_Pyr_H1
SN_Pyr_H21
SN_Pyr_H5
SN_Pyr_H3
SN_Pyr_H5
alp_A_H5
1
alp_A_H11
II.
alp_Bw_H2
alp_Bw_H3
5
4
1
alp_Bw_H1
alp_Be_H4
alp_Be_H2
alp_Be_H3
alp_Be_H1
2
1
1
3
2
3
Distribution of frequences
Frequency
0
5
15
0
10
20
30
40
50
Hamming distance
Pyr_B_H1
Pyr_B_H4
Pyr_B_H2
Pyr_B_H3
1
1
1
2
2
2
