# Supplementary material for: Distribution and population genetic variation of cryptic species of the Alpine mayfly Baetis alpinus (Ephemeroptera: Baetidae) in the Central Alps
Source: BMC Evol Biol. 2016 Apr 12;16:77. doi: 10.1186/s12862-016-0643-y (PMC4828801; doi:10.1186/s12862-016-0643-y)
Supplement: Additional file 6: — Morphological analysis of B. alpinus lineage larval structures. (PDF 8912 kb) [file 12862_2016_643_MOESM6_ESM.pdf]

Additional file 6. Morphological analysis of *B. alpinus* lineage larval structures.

The upper image shows **(a)** the maxilla and the apex of the maxillary palp (black box). The number and distribution of setae at the apex of the maxillary palp is visualized for lineage *A* **(**right: **b** and, left: **c)** and lineage *B* **(**right: **d** and, left: **e)**. The bottom image shows **(f**, **h)** the femora and its dorsal margin (black box). The number of rows of setae on the dorsal margin of the femora is visualized for lineage *A* **(g)** and lineage *B* **(i).**

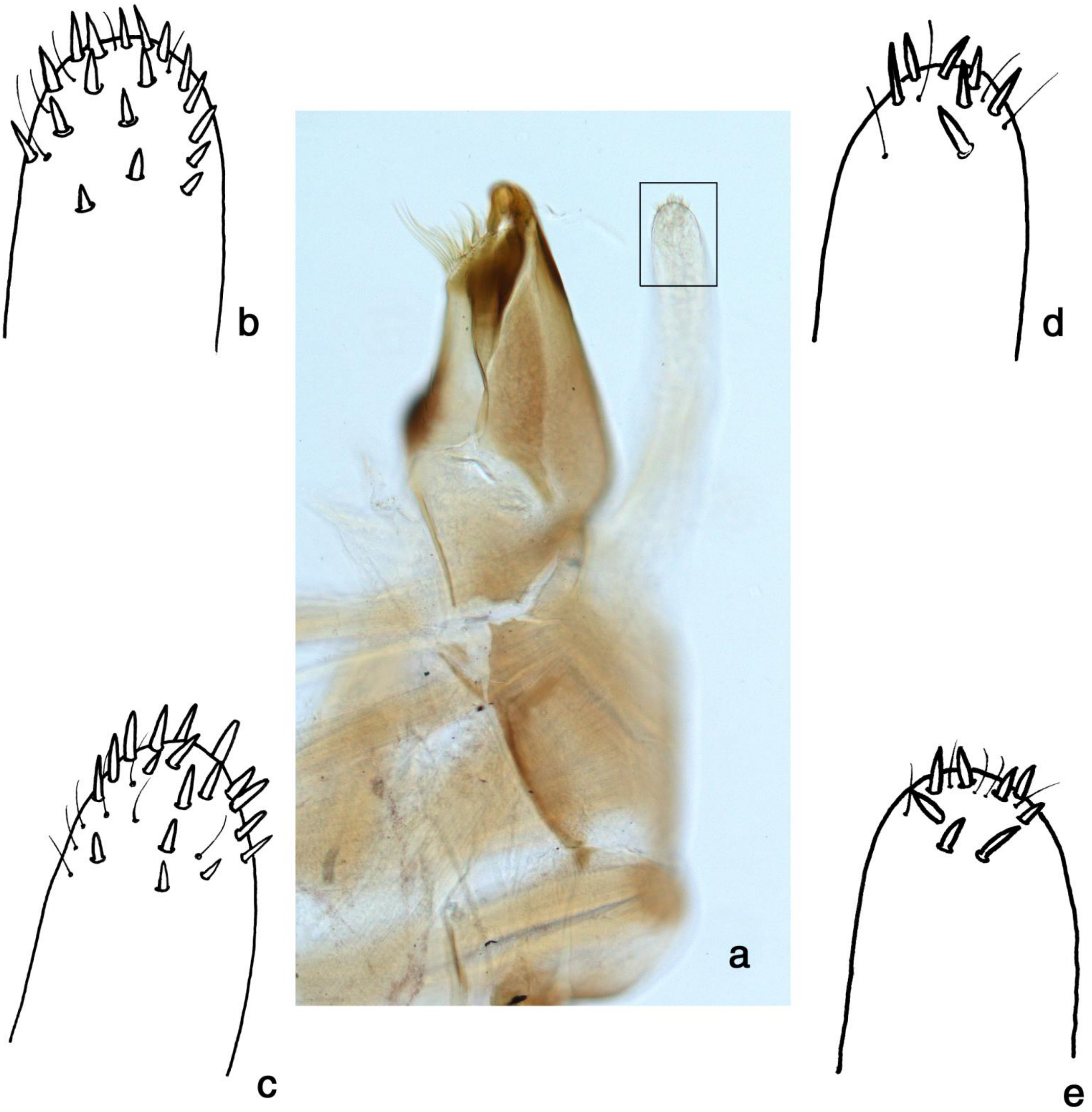

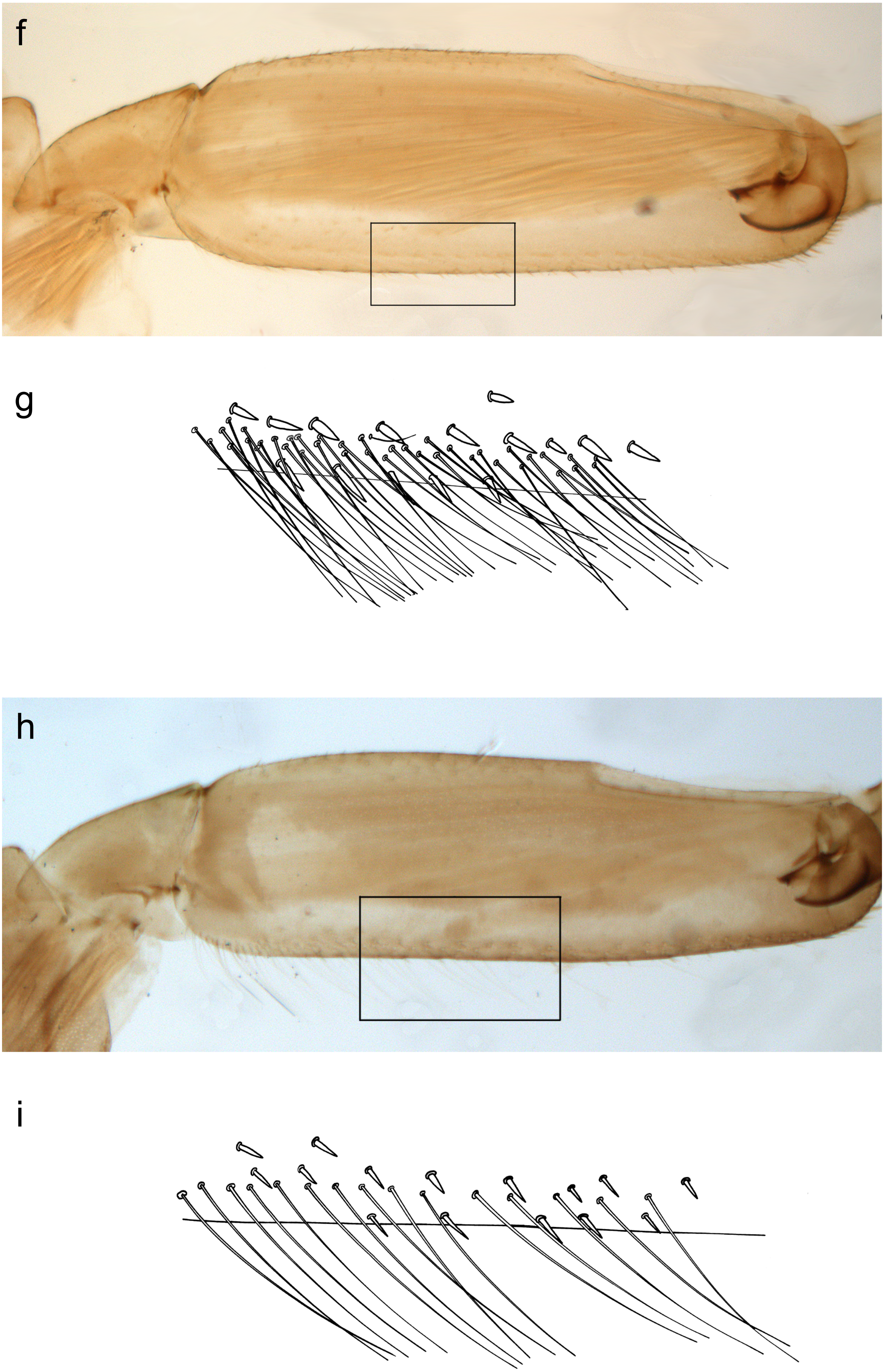
f
g
h
i
